# Dose‐dependent association of systemic comorbidities with periodontitis severity: A large population cross‐sectional study

**DOI:** 10.1002/JPER.25-0055

**Published:** 2025-08-08

**Authors:** Muhammad H. A. Saleh, Hamoun Sabri

**Affiliations:** ^1^ Department of Periodontics and Oral Medicine University of Michigan School of Dentistry Ann Arbor Michigan USA

**Keywords:** comorbidities, humans, periodontal disease, periodontitis, risk factors

## Abstract

**Background:**

To examine whether the associations between periodontitis and multiple systemic conditions increase with increasing severity of periodontitis using a multi‐center electronic health record (EHR) repository.

**Methods:**

A cross‐sectional analysis was conducted using EHR data from 9 dental schools in the United States. We included 264,913 adults (≥18 years) receiving periodontal therapy between 2013 and 2023. Periodontitis severity (no, mild/moderate, severe) was determined, and out of 98 systemic and behavioural conditions, associations with 24 conditions were evaluated using weighted uni‐ and multi‐variate multinomial logistic regressions (with no periodontitis as reference) by 2 adjusted models accounting for age, sex, smoking, and diabetes. Model fit and assumptions were checked using Akaike information criterion (AIC), likelihood ratio test (LRT), false discovery rate (FDR) ‐adjusted *p*‐values, and variance inflation factor (VIF) < 10. The manuscript was prepared following the Oral Health Statistical reporting guidelines (OHStat) and the Strengthening the Reporting of Observational Studies in Epidemiology (STROBE) guidelines.

**Results:**

Among 264,913 adult patients, 166,207 had no periodontitis, and 98,706 had periodontitis. In fully adjusted models of 24 selected systemic comorbidities, the odds ratios (ORs) for severe periodontitis consistently exceeded those for mild/moderate periodontitis. For instance, smoking showed ORs of 1.78 (mild/moderate) versus 5.21 (severe), diabetes (2.20 vs. 5.59), cardiovascular disease (1.53 vs. 2.21), human immunodeficiency virus (HIV) (2.25 vs. 4.07), and Alzheimer's disease (1.84 vs. 3.20). Inverse correlations also strengthened with severity where asthma was 0.80 versus 0.72 in severe compared to mild/moderate periodontitis.

**Conclusions:**

The results of this population‐based study demonstrate that as periodontitis severity escalates, positive and negative associations with systemic conditions become more pronounced.

**Plain language summary:**

This study examined how various chronic health conditions may increase the likelihood of developing more severe gum disease, also known as periodontitis. Using electronic health record data from over 260,000 adults treated at 9 U.S. dental schools, we investigated whether people with systemic diseases—like diabetes, cardiovascular disease, or human immunodeficiency virus (HIV)—were more likely to have mild or severe periodontitis. The results showed that several conditions significantly increased the odds of having more severe gum disease. For instance, individuals with diabetes or cardiovascular problems had more than double the risk of severe periodontitis, while those with HIV or dementia showed even stronger associations. Some conditions, such as asthma or anorexia, were linked to lower odds of gum disease, suggesting that these relationships may be complex and deserve further study. Although this study cannot determine cause and effect, it highlights a pattern: people with chronic diseases are more likely to have more severe gum problems. This emphasizes the need for medical and dental care providers to work together when managing patients with chronic health issues, and it reinforces the importance of oral health as a part of overall well‐being.

## 1 INTRODUCTION

Periodontitis is one of the most common inflammatory conditions in humans posing a significant public health challenge globally; by 2019, the global prevalence had risen dramatically, affecting 1.1 billion individuals,^1^ making periodontal disease more prevalent than cardiovascular disease, which affects 6.6% of the population.^2^ Periodontitis is a multicausal, complex inflammatory condition driven by simultaneous and interacting causal factors. It leads to immunological dysregulation and is compounded by a dysbiotic biofilm, creating a vicious cycle of maladaptive host responses.^3^, ^4^ Periodontitis not only impacts oral health but also has significant implications for general health and well‐being.^5^, ^6^ Traditionally viewed as a localized inflammatory condition, growing evidence over the past 4 decades emphasizes its associations with chronic systemic diseases.^7^ As early as the 1980s, studies have examined links between periodontitis and noncommunicable diseases (NCDs) such as cardiovascular disease, diabetes, and Alzheimer's, many of which are leading causes of death.^8^, ^9^ More evidence has linked periodontitis to other NCDs, such as metabolic diseases, obesity, rheumatoid arthritis, cancers, and respiratory diseases. Monsarrat et al. reported associations with 57 systemic conditions, with over one‐third of periodontal clinical trials exploring these links.^10^

The pro‐inflammatory state common to NCDs also characterizes periodontitis,^11^ suggesting a bidirectional influence between periodontitis and systemic diseases.^11^ This supports the proposition that periodontal disease should be classified as a systemic disease due to its systemic impacts and role as a comorbid condition.^12^ A 2012 expert review concluded that periodontitis contributes to systemic inflammation, influencing the pathophysiology of diabetes, pregnancy complications, and cardiovascular diseases.^13^ Ten years later, a joint workshop held in Spain by European Federation of Periodontology (EFP) and the European arm of the World Organization of Family Doctors (WONCA Europe) came up with the recommendation that “Closer collaboration between oral health professionals and family doctors is important in the early detection and management of NCDs (including periodontitis), and in promoting healthy lifestyles”.^14^ Basically saying, the presence of co‐ factors/indicators, either environmental, behavioral, or biological, increases disease likelihood while their removal decreases it.^15^ Thus, the management of NCDs has evolved substantially, transitioning from medication‐based treatments to more comprehensive approaches that incorporate behavioral interventions to modify risk factors; this essentially has to include the management of periodontitis.^14^ NCDs are affecting a significant portion of the population,^16^ and their exponential rise has led to an increase in individuals with multimorbidity—defined as the presence of 2 or more chronic conditions—now affecting over 20% of the adult population.^17^ Collectively, these NCDs pose a significant burden on healthcare systems globally due to their requirement for long‐term care rather than cures, accounting for approximately 41 million deaths annually (71% of global mortality) and predicted to rise to 52 million deaths by 2030.^18^

Since the degree of inflammation, as well as the bacterial microbiota, are directly correlated with the severity of periodontitis, the correlation between NCDs and periodontitis should be more severe and significant in more severe forms of periodontitis.^13^ These factors impact shared biological mechanisms such as systemic inflammation and immune function, contributing to the onset of NCDs and multimorbidity. Notably, some correlations only emerge with severe periodontitis.^19^ On the flip side of the coin, the response to periodontal therapy is influenced by NCDs.^20^ Periodontal therapy effectively improves local periodontal health and influences systemic biomarkers.^21^, ^22^ Evidence suggests individual responses to periodontal therapy vary, indicating possible treatment‐by‐patient or treatment‐by‐subgroup interactions.^23^, ^24^ This variability reflects the role of individual factors in periodontitis onset and progression following surgical compared to non‐surgical therapy.^25^

Therefore, the aim of this study was to investigate the association between the systemic conditions that have been hypothesized to be related to periodontitis and periodontitis severity using a centralized oral health data repository derived from electronic health record (EHR) data from 9 dental schools in the United States participating in the Consortium of Oral Health Research and Informatics (COHRI).^26^

## 2 MATERIALS AND METHODS

### 2.1 Ethical approval and registration

This study was approved by the University of Michigan Medical School Institutional Review Board (IRBMED) (identifier number: HUM00157260; Amendment: Ame00122603). The 9 participating sites approved this protocol, which was registered by COHRI under project number COHRI‐BM‐DR23. The study was conducted in agreement with the Helsinki Declaration of 1975 (World Medical Association, 1975), as most recently revised in 2013,^27^ and in accordance with the STROBE (Strengthening the Reporting of Observational Studies in Epidemiology) checklist ^28^ and the OHStat (Oral Health Statistical reporting guidelines) checklist.^29^

### 2.2 Setting and study design

The data extraction of this cross‐sectional study, analysis, and reporting are based on a level 2 data access from the centralized dental data repository known as BigMouth.^30^ BigMouth is a dental data repository where 14 participating dental schools in the United States store EHR data bi‐annually. The difference between level 1 and level 2 access in BigMouth is that level 1 access provides summary level data to the researchers. The purpose of level 1 access usually is to provide researchers with information on the type and quantity of data in BigMouth. A researcher could then request level 2 access after the approval of the ethical committee of each of the participating dental schools, which provides detailed row‐level data, which are suitable for statistical analyses. The presented study acquired data from the EHRs of 9 academic institution dental clinics without access to individual patient EHR:

1. University of Texas Health Science Center at Houston, Houston, TX, USA;
2. University of Iowa College of Dentistry and Dental Clinics, Iowa City, IA, USA;
3. Harvard School of Dental Medicine, Boston, MA, USA;
4. Tufts University School of Dental Medicine, Boston, MA, USA;
5. University of California, San Francisco School of Dentistry, San Francisco, CA, USA;
6. University of Colorado School of Dental Medicine, Aurora, CO, USA;
7. Loma Linda University School of Dentistry, Loma Linda, CA, USA;
8. University of Buffalo School of Dental Medicine, Buffalo, NY, USA; and
9. University of Minnesota School of Dentistry, Minneapolis, MN, USA.

All data are stored in a third‐party repository at the University of Texas Health Science Center at Houston (UTHealth Houston) School of Biomedical Informatics. These data are accessed only after pertaining to all patients who fulfilled the eligibility criteria and were seen at registered dental institutions in the United States between January 2013 and June 2023, and updated January 2024.

### 2.3 Participants

The inclusion and exclusion criteria for this study were as follows.

**Inclusion criteria**:

1. Adults with age ≥18 years;
2. The general eligibility criteria for the sample were dentate adult patients receiving either periodontal prophylaxis or periodontal treatment;
3. Periodontal health or gingivitis: This category of patients was determined based on the presence of completed dental terminology (CDT) codes (ADA Current Procedural Terminology)^31^ that resemble treatment—or lack thereof—consistent with periodontal health and/or gingivitis in the respective patient's EHR (e.g., D1110). The CDT coding system ensures consistency in diagnosing and recording periodontitis cases across institutions, minimizing discrepancies in classification. Furthermore, these codes align with established diagnostic criteria in clinical practice, reinforcing the validity of our case definitions;
4. Periodontitis: a category of patients determined based on the presence of completed current CDT codes that resemble non‐surgical and surgical treatment of periodontitis (or stable periodontitis patients) in the respective patient's EHR (e.g., D4341, D4342, D4240, D4241, D4260, D4261, etc.).

**Exclusion criteria**:

1. Edentulous patients;
2. Patients not receiving any periodontal therapy (preventative or therapeutic);
3. Non‐compliant patients. These were defined as patients who fit the inclusion criteria based on our case definition of periodontitis and who have not received any maintenance therapy;
4. Patients with missing data, who were excluded to maintain data integrity and consistency in analysis;

### 2.4 Study sample size

**FIGURE 1:**
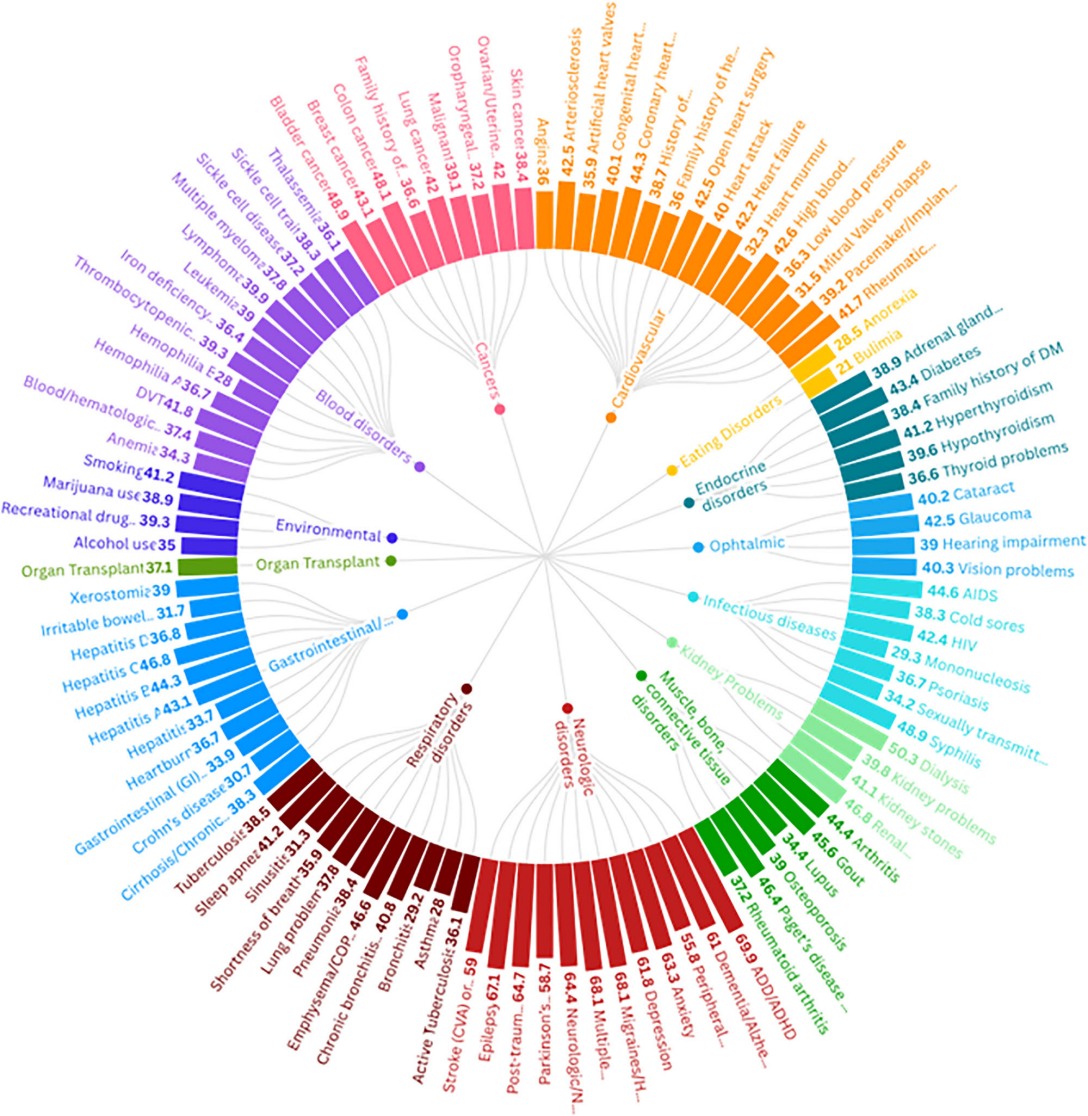
Proportion of periodontitis‐positive cases within each systemic condition group. The percentages indicate the proportion of individuals with each systemic condition who were also diagnosed with periodontitis. For example, a value of 39% for Osteoporosis indicates that 39% of all Osteoporosis cases in the initial cohort had periodontitis.

Figure S1 in online *Journal of Periodontology* presents a flowchart that explains how the study size was arrived at. Briefly, out of approximately 4.6 million individuals in the BigMouth database, 846,668 remained after the preset exclusion criteria. Among these, 558,387 had no periodontitis, and 288,281 had periodontitis. Initially, the periodontitis association was assessed with 98 systemic diseases using a simple chi‐squared test to exclude diseases not associated with periodontitis. Table S1 and Figure 1 in online *Journal of Periodontology* depict the results of the initial analysis on the eligibility of the systemic diseases to be included in the final analysis. The remaining cohort with diseases most associated with periodontitis (24 diseases) made up a total of 264,913 subjects that were included in the final analysis.

### 2.5 Data extraction

A broadly inclusive set of queries was generated to identify count data related to periodontal health and self‐reported systemic disease based on the questionnaire patients are required to fill out at the initial visit and updated every 6–12 months. Additionally, queries regarding any periodontal therapy that was provided were collected. The queries, based on CDT codes^32^ in‐patient EHRs, are outlined in Figure S2 in online *Journal of Periodontology*.

Patients were stratified into 3 groups based on the therapy provided:

- No‐periodontitis group: Only periodontal prophylaxis and no periodontal active therapy was provided;
- Mild/moderate periodontitis: Patients receiving only non‐surgical therapy with or without local antimicrobial therapy; and
- Severe periodontitis group: Patients receiving non‐surgical AND surgical therapy (resective or regenerative) OR Patients receiving > 4 quadrants of non‐surgical therapy (repeated non‐surgical therapy) with or without local antimicrobial therapy.

### 2.6 Statistical analysis

Descriptive statistics were calculated for the full patient cohort and stratified by no periodontitis, mild/moderate periodontitis, and severe periodontitis groups. Continuous variables, such as age, were reported as means and standard deviations (SD), while categorical variables, including systemic diseases and other covariates, were presented as counts and percentages. To investigate the association between each independent variable and periodontitis, a weighted univariate multinomial logistic regression was performed, with the No periodontitis group serving as the reference category. Weighting was applied to account for potential imbalance in the group sizes among the 3 periodontitis categories ensuring that the smaller severe group was appropriately represented in the analysis and reducing potential bias due to this unequal distribution.

Following the unadjusted model (model 1) 2 more regression models were employed to adjust for possible confounders. Model 2 was adjusted to age and sex of the subjects, while in model 3 in addition to these variables, smoking and diabetes were also included (known risk factors of periodontitis). A weighted multivariate multinomial logistic regression was subsequently carried out for these 2 models. Model fit was assessed using the Akaike information criterion (AIC) and the likelihood ratio test (LRT) to compare the full model to a null model. Weighting was maintained for consistency with the univariate model. To adjust for multiple comparisons, *p*‐values were adjusted using the false discovery rate (FDR) method. Furthermore, multicollinearity among independent variables was assessed using the variance inflation factor (VIF), with a threshold of 10 indicating significant multicollinearity. In the final model, all VIF values were below this threshold, suggesting no significant multicollinearity issues. All statistical analyses were performed by 1 author with experience in biostatistics (H.S.) using statistical software,* and multiple statistical packages† were used for the analyses.

## 3 RESULTS

### 3.1 Descriptive results and characteristics of the included patient cohort

The study cohort included a total of 264,913 subjects, distributed across 3 main groups categorized by their periodontitis status: 166,207 (62.7%) subjects with no periodontitis, 97,706 with periodontitis (37.3%). Figure 1 in online *Journal of Periodontology* depicts the prevalence of systemic diseases association between periodontitis and 99 systemic diseases was assessed using a simple chi‐squared test to exclude diseases not associated with periodontitisin periodontitis versus no periodontitis patients in the initial cohort.

For severity, the mean age of the participants increased with disease severity, from 42.52 years (± 14.87) in the no‐periodontitis group to 51.87 years (± 14.68) and 52.98 years (± 12.82) in the mild/moderate and severe periodontitis groups, respectively. With regard to sex of the subjects, females comprised 57.61% of the no‐periodontitis group, 48.87% of the mild/moderate group, and 48.26% of the severe periodontitis group.

**FIGURE 2:**
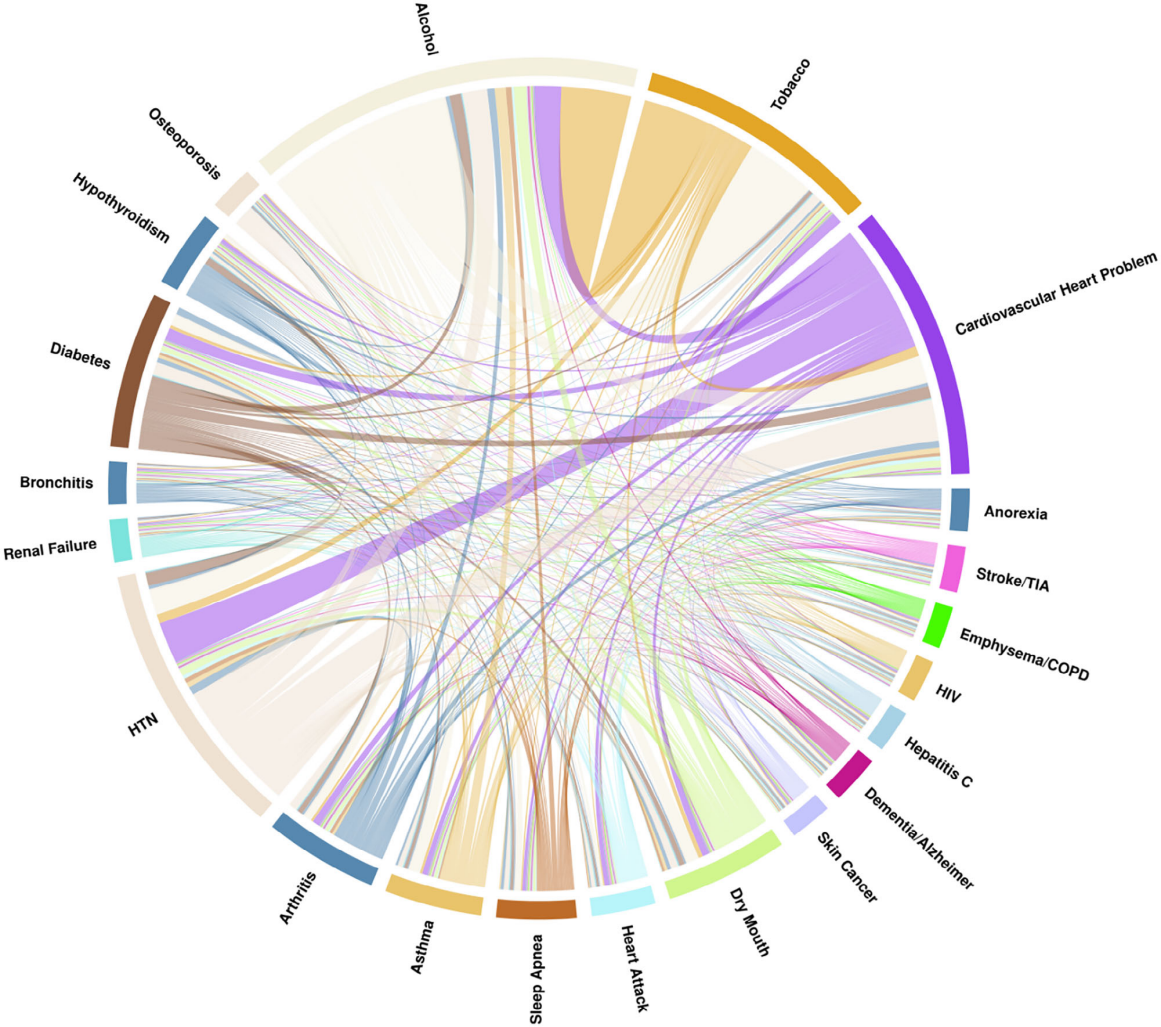
Chord diagram illustrating the co‐occurrence of systemic diseases in healthy individuals. The chord thickness represents the proportion of individuals with overlapping systemic conditions, and the segment size reflects the overall prevalence of each disease in this group

**FIGURE 3:**
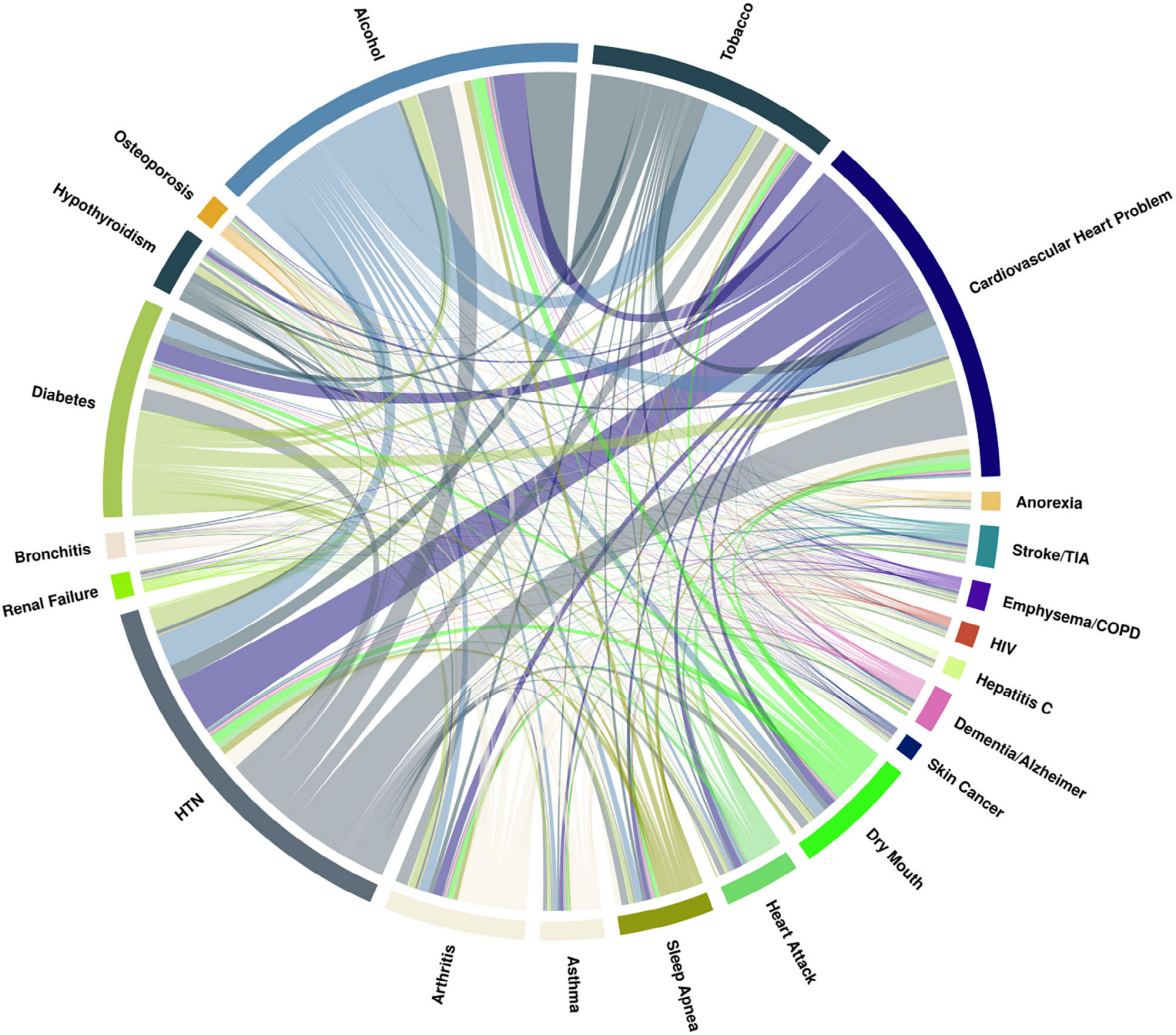
Chord diagram representing the co‐occurrence of systemic diseases in individuals with mild/moderate periodontitis. The width of each chord corresponds to the percentage of cases where 2 diseases co‐occur, while the arc length around the circle indicates the overall prevalence of each disease

**FIGURE 4:**
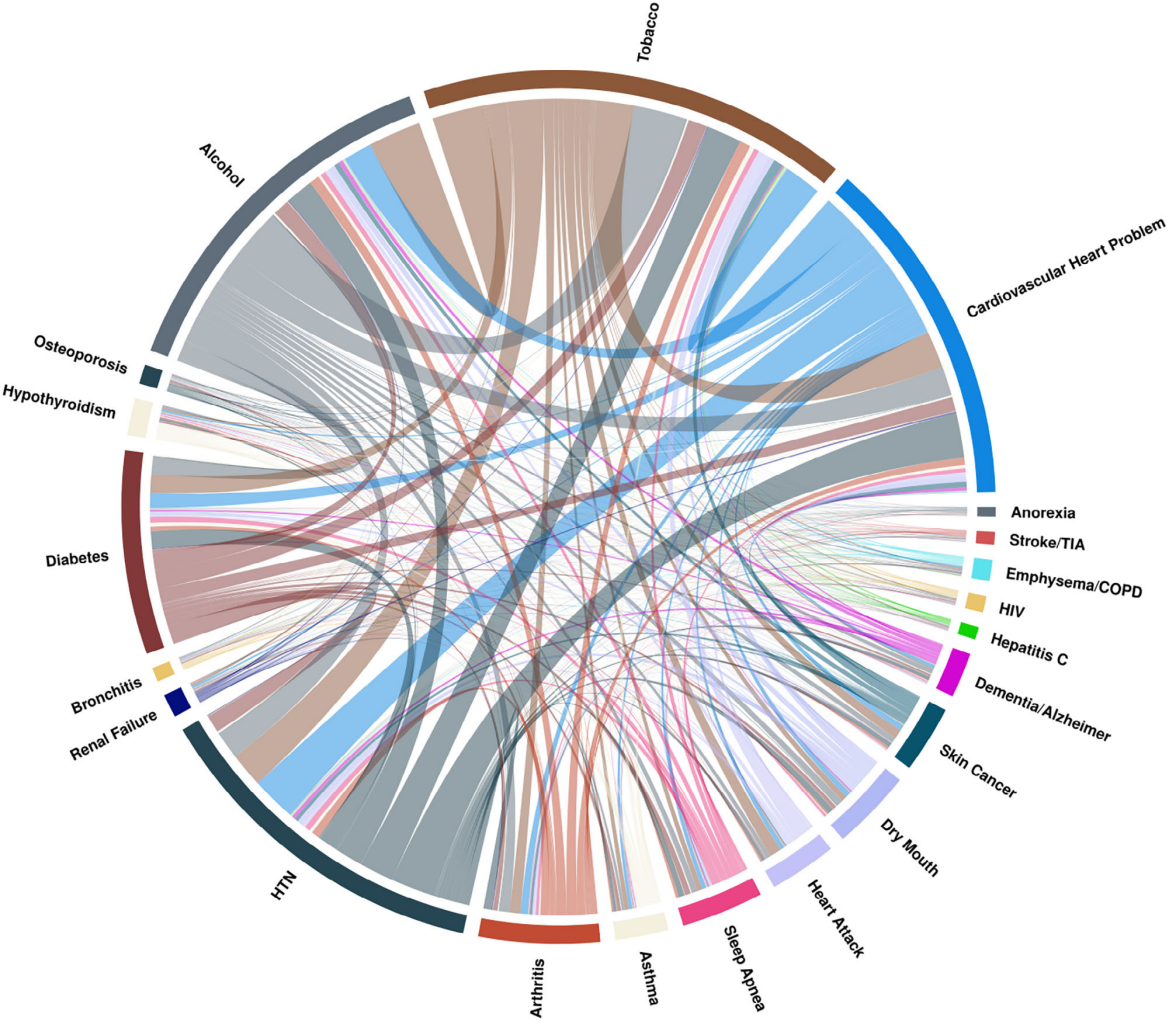
Chord diagram depicting the co‐occurrence of systemic diseases in individuals with severe periodontitis. The chord width indicates the strength of co‐occurrence between diseases, while the arc length represents the prevalence of each condition in the population

According to the main trend, the prevalence of comorbidities and risk factors was higher in the periodontitis groups. Alcohol consumption and tobacco use were reported in 35.9% and 12.9% of the no‐periodontitis group, compared to 40.8% and 22.5% in the severe periodontitis group. Cardiovascular problems were present in 5.72% of the no‐periodontitis group, increasing to 15.9% and 25.8% in the mild/moderate and severe groups, respectively. Other systemic conditions, including diabetes, hypertension (HTN), dry mouth, and dementia/Alzheimer's disease, were more common in individuals with severe periodontitis. Table 1 provides the full results of the descriptive analysis and patient characteristics. Figures 2, 3, 4 depict the proportion of the total prevalence of each systemic condition and the co‐occurrence of each with other conditions as chord graphs.

**TABLE 1:** Descriptive statistics of included patient cohort

Variable	No periodontitis	Mild/moderate periodontitis	Severe periodontitis
Total count (N)	166207	96123	1583
Age (mean ±SD)	42.52 ± 14.87	51.87 ± 14.68	52.98 ± 12.82
Sex (female; %)	95758 (57.61%)	46982 (48.87%)	764 (48.26)
Alcohol	59619 (35.9%)	35820 (37.3%)	646 (40.8%)
Tobacco	21443 (12.9%)	21583 (20.7%)	327 (22.5%)
Drugs	23459 (14.1%)	11713 (12.2%)	186 (11.7%)
Cardiovascular/heart problem	9500 (5.72%)	15281 (15.9%)	408 (25.8%)
Bronchitis	342 (0.206%)	501 (0.521%)	5 (0.316%)
Diabetes	4578 (2.75%)	8605 (8.95%)	200 (12.6%)
Hypothyroidism	1608 (0.967%)	1935 (2.01%)	30 (1.90%)
Osteoporosis	534 (0.321%)	598 (0.622%)	16 (1.01%)
Arthritis	2748 (1.65%)	4809 (5.00%)	110 (6.95%)
Depression	1451 (0.873%)	1115 (1.16%)	16 (1.01%)
HTN	8851 (5.33%)	15948 (16.6%)	403 (25.5%)
Renal failure/Insufficiency	220 (0.132%)	440 (0.458%)	15 (0.948%)
Dry mouth	4066 (2.45%)	5192 (5.40%)	112 (7.08%)
Heart attack	962 (0.579%)	1943 (2.02%)	22 (1.39%)
Sleep apnea	1784 (1.07%)	3055 (3.18%)	82 (5.18%)
Asthma	2801 (1.69%)	2478 (2.58%)	59 (3.73%)
Anorexia	62 (0.037%)	13 (0.013%)	0 (0%)
Stroke/TIA	333 (0.2%)	795 (0.827%)	5 (0.316%)
Emphysema/COPD	281 (0.169%)	536 (0.558%)	14 (0.884%)
HIV	277 (0.167%)	470 (0.489%)	17 (1.07%)
Hepatitis C	125 (0.075%)	228 (0.237%)	10 (0.632%)
Dementia/Alzheimer	747 (0.449%)	1126 (1.17%)	44 (2.78%)
Endocarditis	21 (0.012%)	28 (0.029%)	0 (0%)
Skin cancer	103 (0.062%)	143 (0.149%)	6 (0.379%)

Abbreviations: COPD, chronic obstructive pulmonary disease; HIV, human immunodeficiency virus; HTN, hypertension; TIA, transient ischemic attack.

### 3.2 Uni‐ and multivariate regression analysis

**FIGURE 5:**
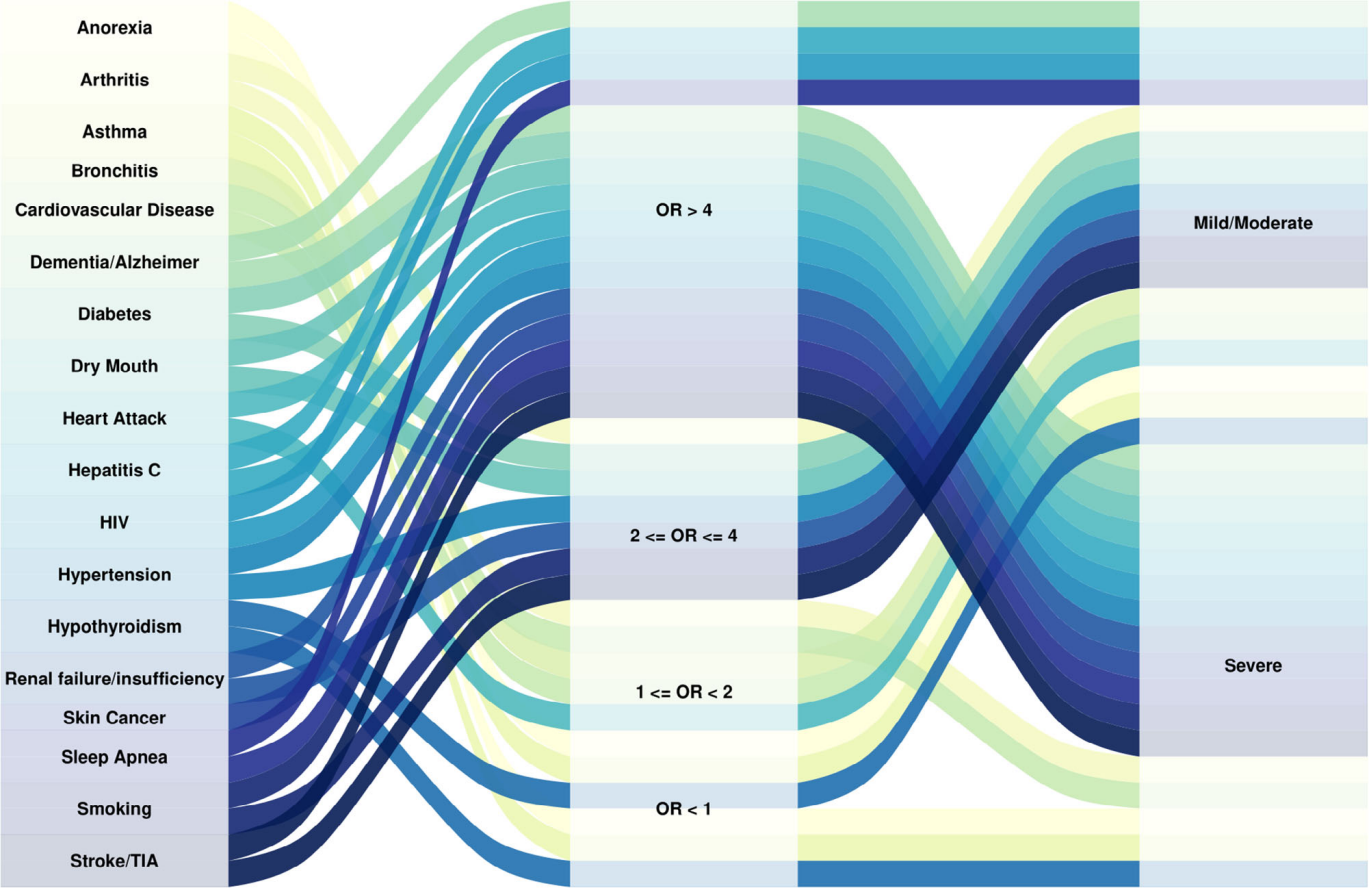
Sankey diagram illustrating the associations between systemic diseases, odds ratio (OR) ranges, and periodontitis severity (mild/moderate and severe). The width of each connecting band represents the presence and strength of the association, with odds ratio (OR) ranges categorized as OR > 4, 2 < OR ≤ 4, 1 < OR < 2, and OR < 1. Color coding corresponds to specific systemic diseases. The OR ratios in this figure are based on Model 3

Figure 5 depicts the significant associations between the systemic covariates and periodontitis severity.

#### 3.2.1 Model 1 (Unadjusted analysis)

The unadjusted regression analysis (Model 1) identified significant associations between various demographic, lifestyle, and systemic factors and the likelihood of developing mild/moderate and severe periodontitis. Key predictors included age, male sex, smoking, diabetes, and cardiovascular disease, with additional contributions from conditions such as hypothyroidism, arthritis, dementia, and skin cancer. Detailed results are provided in the Supplementary Material.

#### 3.2.2 Model 2 (adjusted for age and sex)

See [Supplementary Material] for this section.

#### 3.2.3 Model 3 (adjusted for age, sex, smoking, and diabetes)

The third regression model, adjusted for age, sex, smoking, and diabetes, provided an assessment of the associations between systemic conditions and periodontitis severity while controlling for known risk factors. Cardiovascular disease remained a significant predictor, with adjusted odds ratios (ORs) of 1.53 (95% confidence interval [CI]: 1.48–1.57, *p* < 0.001) for mild/moderate periodontitis and 2.21 (95% CI: 1.94–2.53, *p* < 0.001) for severe periodontitis. While bronchitis retained significance for mild/moderate periodontitis (OR: 1.41, 95% CI: 1.22–1.63, *p* < 0.001), it became non‐significant for severe periodontitis (*p* = 0.20). Stroke/transient ischemic attack (TIA) remained significant for mild/moderate periodontitis (OR: 1.66, 95% CI: 1.45–1.90, *p* < 0.001) but lost significance for severe (*p* = 0.053).

Arthritis maintained its association, with adjusted ORs of 1.51 (95% CI: 1.43–1.58, *p* < 0.001) for mild/moderate and 1.54 (95% CI: 1.25–1.90, *p* < 0.001) for severe periodontitis. HTN showed a consistent association across both severity levels, with ORs of 1.73 (95% CI: 1.67–1.78, *p* < 0.001) for mild/moderate and 2.27 (95% CI: 1.99–2.61, *p* < 0.001) for severe periodontitis. Renal failure/insufficiency remained significant, with ORs of 1.52 (95% CI: 1.28–1.81, *p* < 0.001) for mild/moderate and 2.14 (95% CI: 1.26–3.64, *p* = 0.005) for severe periodontitis.

Dry mouth continued to exhibit significant associations, with ORs of 1.58 (95% CI: 1.51–1.66, *p* < 0.001) for mild/moderate and 1.62 (95% CI: 1.32–1.98, *p* < 0.001) for severe periodontitis. Heart attack was associated with higher odds of periodontitis, with ORs of 1.31 (95% CI: 1.20–1.42, *p* < 0.001) for mild/moderate and 2.82 (95% CI: 2.24–3.56, *p* < 0.001) for severe periodontitis. Sleep apnea also remained strongly associated with both mild/moderate (OR: 1.88, 95% CI: 1.77–2.00, *p* < 0.001) and severe periodontitis (OR: 2.27, 95% CI: 1.79–2.87, *p* < 0.001).

Asthma showed a protective association after full adjustment, with ORs of 0.80 (95% CI: 0.75–0.85, *p* < 0.001) for mild/moderate and 0.72 (95% CI: 0.53–0.99, *p* = 0.041) for severe periodontitis. Anorexia also demonstrated reduced odds for both mild/moderate (OR: 0.41, 95% CI: 0.22–0.77, *p* = 0.006) and severe periodontitis (OR: 0.0016, 95% CI: 0.0016–0.0016, *p* < 0.001).

HIV and hepatitis C were both significantly associated with increased odds of periodontitis, with HIV showing ORs of 2.25 (95% CI: 1.92–2.63, *p* < 0.001) for mild/moderate and 4.07 (95% CI: 2.46–6.71, *p* < 0.001) for severe periodontitis. Hepatitis C demonstrated ORs of 1.88 (95% CI: 1.50–2.36, *p* < 0.001) for mild/moderate and 3.62 (95% CI: 1.87–6.99, *p* < 0.001) for severe periodontitis. Dementia/Alzheimer's disease remained strongly associated, with ORs of 1.84 (95% CI: 1.67–2.03, *p* < 0.001) for mild/moderate and 3.20 (95% CI: 2.33–4.39, *p* < 0.001) for severe periodontitis.

Finally, skin cancer retained its high association with severe periodontitis (OR: 31.64, 95% CI: 22.79–43.93, *p* < 0.001) but was not significant for mild/moderate (*p* = 0.46). These results underscore the interplay between systemic conditions and periodontitis severity, with specific associations reflecting significant attenuation or reversals following multivariable adjustment.

## 4 DISCUSSION

See Supplementary Materials in online *Journal of Periodontology* for additional information regarding Section 4.

This cross‐sectional study explored the associations between various systemic conditions and periodontitis severity in a large population of approximately a quarter of a million individuals. Our results overall demonstrated that as periodontitis severity escalates, the associations with systemic conditions become more pronounced. We used 3 models: (a) none adjusted; (b) adjusted for age, sex; and (c) adjusted for age, sex, smoking, and diabetes. As expected, with controlling more confounders, some associations were lost. Yet almost half of the associations‐maintained association, and a handful maintained it with OR ≥ 3 (Table 2).

**TABLE 2:** The results of the three regression models used in this study on the outcome of periodontitis severity

	Model 1^a^			Model 2^b^			Model 3^c^		
	Periodontitis category			Periodontitis category			Periodontitis category		
Variable	Healthy	Mild/moderate	Severe	Healthy	Mild/moderate	Severe	Healthy	Mild/moderate	Severe
Age	Ref.	1.04 [1.04–1.04], *p* < 0.0001	(1.05 [1.04–1.05]), *p* < 0.0001	Ref.	‐	‐	Ref.	‐	‐
Sex (M)	Ref.	(1.42 [1.40–1.44]), *p* < 0.0001	(1.46 [1.32–1.61]), *p* < 0.0001	Ref.	‐	‐	Ref.	‐	‐
Tobacco	Ref.	(1.95 [1.91–1.99]), *p* < 0.0001	(5.69 [5.15–6.29]), *P* < 0.0001	Ref.	1.78 [1.74–1.82], *p* < 0.001	**5.21** [4.71–5.76], *p* < 0.001	Ref.	‐	‐
Alcohol	Ref.	(1.06 [1.04–1.08]), *p* < 0.0001	(1.23 [1.11–1.36]), *p* = 0.00005	Ref.	1.07 [1.05–1.09], *p* < 0.001	1.24 [1.12–1.37], *p* < 0.01	Ref.	0.989 (0.972–1.007), *p* = 0.2206	0.935 (0.843–1.037), *p* = 0.2061
Cardiovascular Disease	Ref.	3.12 [3.03–3.20], *p* < 0.0001	5.73 [5.11–6.42], *p* < 0.0001	Ref.	1.74 [1.69–1.79], *p* < 0.001	3.26 [2.88–3.69], *p* < 0.001	Ref.	1.525 (1.480–1.572), *p*<0.001	2.214 (1.939–2.528), *p*<0.001
Bronchitis	Ref.	2.54 [2.21–2.92], *p* < 0.0001	1.54 [0.63–3.72], *p* = 0.341	Ref.	1.72 [1.49–1.99], *p* < 0.001	1.00 [0.41–2.43], *p* = 0.99	Ref.	1.409 (1.216–1.634), *p*<0.001	0.553 (0.226–1.357), *p* = 0.1958
Diabetes	Ref.	3.47 [3.35–3.60], *p* < 0.0001	8.76 [7.72–9.95], *p* < 0.0001	Ref.	2.20 [2.12–2.29], *p* < 0.001	**5.59** [4.90–6.39], *p* < 0.001	Ref.	‐	‐
Hypothyroidism	Ref.	2.10 [1.97–2.25], *p* < 0.0001	1.98 [1.37–2.85], *p* = 0.00025	Ref.	1.45 [1.35–1.56], *p* < 0.001	1.31 [0.91–1.90], *p* = 0.15	Ref.	0.838 (0.776–0.905), *p*<0.001	0.372 (0.254–0.544), *p*<0.001
Osteoporosis	Ref.	1.94 [1.73–2.18], *p* < 0.0001	3.17 [1.93–5.23], *p* < 0.0001	Ref.	1.06 [0.94–1.19], *p* = 0.37	1.64 [0.99–2.71], *p* = 0.05	Ref.	0.979 (0.867–1.106), *p* = 0.7333	1.387 (0.833–2.310), *p* = 0.2083
Arthritis	Ref.	3.13 [2.99–3.29], *p* < 0.0001	4.50 [3.70–5.48]), *p* < 0.0001	Ref.	1.75 [1.66–1.84], *p* < 0.001	2.37 [1.94–2.91], *p* < 0.001	Ref.	1.505 (1.430–1.584), *p*<0.001	1.539 (1.248–1.897), *p*<0.001
Hypertension	Ref.	3.54 [3.44–3.63], *p* < 0.0001	6.08 [5.42–6.82], *p* < 0.0001	Ref.	1.97 [1.91–2.03], *p* < 0.001	3.41 [3.01–3.87], *p* < 0.001	Ref.	1.726 (1.674–1.779), *p*<0.001	2.274 (1.986–2.605), *p*<0.001
Renal failure/insufficiency	Ref.	3.47 [2.95–4.08], *p* < 0.0001	7.22 [4.27–12.21]), *p* < 0.0001	Ref.	1.99 [1.68–2.35], *p* < 0.01	3.93 [2.32–6.67], *p* < 0.01	Ref.	1.523 (1.281–1.811), *p*<0.001	2.143 (1.261–3.642), *p* = 0.0049
Dry mouth	Ref.	2.28 [2.18–2.37], *p* < 0.0001	3.04 [2.50–3.69]), *p* < 0.0001	Ref.	1.78 [1.71–1.86], *p* < 0.001	2.32 [1.91–2.82], *p* < 0.001	Ref.	1.582 (1.513–1.655), *p*<0.001	1.620 (1.324–1.982), *p*< 0.001
Heart Attack	Ref.	3.54 [3.28–3.83], *p* < 0.0001	10.57 [8.48–13.17]), *p* < 0.0001	Ref.	1.59 [1.47–1.72], *p* < 0.001	4.61 [3.67–5.78], *p* < 0.001	Ref.	1.306 (1.203–1.417), *P*<0.001	2.824 (2.238–3.564), *p*<0.001
Sleep apnea	Ref.	3.03 [2.85–3.21], *p* < 0.0001	5.03 [4.01–6.32]), *p* < 0.0001	Ref.	2.17 [2.04–2.31], *p* < 0.001	3.52 [2.80–4.43], *p* < 0.001	Ref.	1.880 (1.766–2.002), *p*<0.001	2.266 (1.791–2.869), *p*<0.001
Asthma	Ref.	1.03 [0.97–1.08], *p* = 0.328	1.25 [0.92–1.70]), *p* = 0.152	Ref.	0.91 [0.86–0.97], *p* = 0.002	1.10 [0.81–1.49], *p* = 0.54	Ref.	0.799 (0.754–0.847), *p*<0.001	0.722 (0.529–0.987), *p* = 0.0411
Anorexia	Ref.	0.36 [0.20–0.66]), *p* = 0.00088	7.7e‐05 [7.7e‐05–7.7e‐05]), *p* < 0.0001	Ref.	0.49 [0.26–0.91], *p* = 0.02	0.03 [0.03–0.03], *p* < 0.001	Ref.	0.407 (0.215–0.772), *p* = 0.0059	0.0016 (0.0016–0.0016), *p*<0.001
Stroke/TIA	Ref.	4.15 [3.65–4.72], *p* < 0.0001	1.58 [0.65–3.82]), *p* = 0.312	Ref.	2.07 [1.82–2.37], *p* < 0.00	0.74 [0.30–1.79], *p* = 0.50	Ref.	1.663 (1.454–1.901), *p*<0.001	0.416 (0.171–1.012), *p* = 0.0532
Emphysema/COPD	Ref.	3.31 [2.87–3.83], *p* < 0.0001	5.30 [3.10–9.08]), *p* < 0.0001	Ref.	1.55 [1.33–1.79], *p* < 7.53e‐09	2.31 [1.34–3.97], *p* = 0.02	Ref.	1.161 (0.998–1.350), *p* = 0.0525	1.010 (0.583–1.749), *p* = 0.9730
HIV	Ref.	2.94 [2.54–3.41], *p* < 0.0001	6.50 [3.98–10.64]), *p* < 0.0001	Ref.	2.48 [2.12–2.90], *p* < 0.00	5.42 [3.30–8.88], *p* < 0.001	Ref.	2.247 (1.921–2.628), *p*<0.001	**4.065** (2.463–6.709), *p*<0.001
Hepatitis C	Ref.	3.16 [2.54–3.94], *p* < 0.0001	8.26 [4.30–15.86]), *p* < 0.0001	Ref.	2.18 [1.75–2.73], *p* < 8.59e‐12	5.69 [2.97–10.89], *p* < 0.001	Ref.	1.883 (1.500–2.364), *p*<0.001	**3.615** (1.871–6.986), *p*<0.0001
Dementia/Alzheimer	Ref.	2.63 [2.39–2.88], *p* < 0.0001	6.33 [4.65–8.62]), *p* < 0.0001	Ref.	2.16 [1.96–2.38], *p* < 0.00	5.12 [3.75–6.98], *p* < 0.001	Ref.	1.840 (1.666–2.032), *p*<0.001	**3.200** (2.330–4.394), *p*<0.001
Skin Cancer	Ref.	2.40 [1.86–3.10], *p* < 0.0001	74.61 [54.85–101.50], *p* < 0.0001	Ref.	1.18 [0.91–1.52], *p* = 0.22	35.46 [25.84–48.66], *p* < 0.001	Ref.	1.103 (0.849–1.432), *p* = 0.4640	**31.644** (22.794–43.931), *p*<0.001

*Note*: All the *p* values from models 2 and 3 were calculated taking into account the false discovery rate (FDR); for example, FDR *p* values. All the *p* values from models 2 and 3 were calculated taking into account the FDR; for example, FDR *p* values.

Abbreviations: COPD, chronic obstructive pulmonary disease; TIA, transient ischemic attack.

^a^ Model 1: Raw univariate multinomial logistic regression analysis without adjustments for any covariates.

^b^ Model 2: The model was adjusted for age and sex covariates. (multivariable multinomial logistic regression).

^c^ Model 3: The model was adjusted for age, sex, smoking, and diabetes covariates (multivariable multinomial logistic regression).

Conducting longitudinal studies to establish causality and identify risk factors for periodontitis is often challenging and not always feasible. In such cases, cross‐sectional and retrospective studies provide valuable initial insights and can serve as pilot data for future longitudinal research. Importantly, studies with large sample sizes are essential for enhancing the generalizability of findings. Recognizing this, our analysis leveraged a large dataset (BigMouth)^33^ to provide a more comprehensive and generalizable assessment of systemic conditions within the context of periodontitis.

Other studies also used BigMouth, mostly using level‐1 data. Chatzopoulos et al.,^34^ assessed 1985 subjects using BigMouth data and found negative association of diabetes and kidney disease with periodontitis. A similar study by the same group on 2069 individuals resulted in significant association between smoking status and grade C periodontitis while no other remarkable associations reported.^35^ In general, the main discrepancy in other BigMouth studies that made their results unreliable was its dependence on diagnosis made by students in the EHR. Agreeing on a diagnosis using 2017 world workshop demonstrated moderate concordance (*K* value: 0.49 for Stage and 0.50 for Grade) even among world expert clinicians with select cases.^36^ Most periodontal diagnoses in EHR systems in the United States are done by undergraduate students. On the flip side, for any therapy/intervention to be approved, a specialized clinical instructor or school professor/similar needs to approve the treatment after a thorough inspection. This also highlights 1 of the limitations of this study. Relying on treatment codes has certainly led to selection bias, where patients who refused treatment or required treatment but could not afford it were not included in the study. Nonetheless, the prevalence of periodontitis in the present study (37.3%) was very close to other US population studies reporting a prevalence of 42.2%.^37^ As noted by the authors of an EFP/APP consensus on the standards for reporting chronic periodontitis prevalence and severity: “it appears unlikely that a single ‘case definition’ will suit all purposes (including estimates of prevalence, severity, treatment needs, identification of risk factors and disease activity)”.^38^

A notable trend observed in our findings is that the strength of the association between periodontitis and systemic diseases often increases with the severity of periodontitis. For example, conditions like smoking, diabetes, cardiovascular disease, and dementia showed progressively stronger associations as periodontitis transitioned from mild/moderate to severe forms.

In contrast to the systemic conditions discussed above, several other factors exhibited a negative association with periodontitis categories. Hypothyroidism (mild‐moderate OR: 0.838 [0.776–0.905], severe OR: 0.372 [0.254–0.544], *p* < 0.001), asthma (mild‐moderate OR: 0.799 [0.754–0.847], *p* < 0.001, severe OR: 0.722 [0.529–0.987], *p* = 0.0411), and anorexia (mild‐moderate OR: 0.407 [0.215–0.772], *p* = 0.0059, severe OR: 0.0016 (0.0016–0.0016), *p* < 0.001) were among these conditions. Several recent studies have investigated the possible relationship between periodontitis and thyroid function.^39^, ^40^, ^41^, ^42^ Within these studies, when considering risk of periodontitis in hypothyroid patients, generally the evidence is inconclusive. While studies by Ortarzewska et al.,^43^ and Al Ahmari et al.,^40^ suggest a positive association and worse periodontal health parameters in subjects with hypothyroidism, a recent scoping review evaluating 29 articles suggest very limited evidence and mixed reports on either no or positive association between the 2 conditions.^41^ The negative association observed in our study between hypothyroidism and periodontitis may reflect unique characteristics of our dataset or underlying methodological differences. It is possible that individuals with hypothyroidism in our cohort were under medical care, which may have included better overall health monitoring and oral health awareness, indirectly reducing their periodontitis risk. Alternatively, this finding could be influenced by confounding factors not fully accounted for, such as differences in sample composition or healthcare access. Given the mixed evidence in the literature and the contrasting findings in our analysis, further research is warranted to clarify the nature of this relationship, ideally through longitudinal studies with comprehensive control for confounding variables.

When focusing on asthma, there are 2 directions in the current evidence, suggesting either a positive^44^, ^45^, ^46^ or negative^19^, ^47^, ^48^ association with periodontitis. We argue that discrepancy in most cases is due to the case definition of periodontitis and the outcomes measures utilized. The protective effect of asthma on periodontitis has been indicated by a case–control using Stage, Grade, and periodontal tooth loss as outcomes (OR = 0.10, *p* < 0.001),^47^ an NHANES cohort with (OR = 0.51, 95% CI = 0.30–0.87) and a 2‐sample mendelian randomization analysis (OR = 0.34; 95% CI = [0.132,0.87]; *p* = 0.025). Pinto and co‐workers, for instance, defined a periodontitis case as “patients presenting with 4 or more teeth, each having 1 or more sites with a probing depth of 4 mm or greater, clinical attachment loss of 3 mm or greater at the same site, and presence of bleeding upon stimulation”. Their outcomes were the metagenomics of subgingival biofilms in individuals with varying degrees of asthma.^45^ Briefly, the suggested mechanism underlying this is suggested to be that asthma might influence the immune response in a manner protective against periodontitis. Specifically, the shift toward a Th2‐dominated immune response in asthma can lead to reduced Th1‐mediated inflammation, associated with tissue destruction in periodontitis. Moreover, altered cytokine profiles, such as decreased interleukin‐1 beta (IL‐1β) and tumor necrosis factor alpha (TNF‐α), may play a role in mitigating the severity of periodontal disease. This inverse association has been further supported by population‐based studies demonstrating that individuals diagnosed with asthma exhibit a lower prevalence of periodontitis compared to the general population^48^. Additionally, Rivera et al.^49^ reported that asthma patients using medication had even lower odds of severe periodontitis (OR = 0.20, 95% CI: 0.09–0.43), supporting the hypothesis that chronic microbial stimulation in periodontitis may foster immune tolerance, reducing susceptibility to atopic conditions like asthma.

The observed inverse association between anorexia and periodontitis should be interpreted with caution due to its statistical limitations. Notably, the model assessing this relationship exhibited 1 of the lowest performances across all regressions, with high AIC/BIC values and a McFadden's *R* ^2^ at the lower end of the reported range. Furthermore, the severe periodontitis group contained no recorded cases of anorexia, limiting statistical power and increasing the likelihood of bias. Given these constraints, the inverse association may be a reflection of data sparsity rather than a genuine protective effect. Future studies with larger sample sizes and more robust modeling approaches are needed to clarify this relationship. While anorexia is well established as a risk factor for gingival recession, evidence linking it directly to periodontitis remains very limited, primarily associating its impact with behavioral and environmental factors rather than intrinsic periodontal pathology.^49^, ^50^, ^51^ A recent review noted that individuals with anorexia nervosa often engage in intense and frequent toothbrushing to obscure the oral consequences of vomiting. Additionally, most studies included in this review reported that these patients exhibit superior oral hygiene practices. Collectively, these findings indicate that this population may confer a protective effect against periodontal disease.^50^

The exceptionally large sample size of this study offers a first‐of‐its‐kind analysis and allows for increased statistical power, enabling the detection of associations that might otherwise go unnoticed in smaller studies. However, it may be so large that it flags every tiny difference as “significant.” The reader should thus examine the effect size carefully (Table 2). The reliance on secondary data introduces potential limitations related to selection bias, variability in data collection methods across clinical centers, and binary disease outcomes, which oversimplify conditions without accounting for their severity or progression. Additionally, self‐reported systemic conditions may lead to underreporting, particularly for asymptomatic or undiagnosed cases.

The study's cross‐sectional design precludes establishing temporal or causal relationships, limiting the ability to infer whether systemic conditions contribute to periodontitis or vice versa. Furthermore, the absence of detailed information on confounding factors, such as oral hygiene practices, may influence the observed associations, and residual confounding cannot be ruled out. While the dataset's size enhances generalizability, its clinical origin may result in a population skewed toward individuals actively seeking dental care, which could limit applicability to broader populations.

## 5 CONCLUSIONS

As periodontitis severity increased from no periodontitis to mild/moderate to severe, the strength of association with numerous comorbidities—most notably diabetes, cardiovascular disease, HIV, and Alzheimer's disease—became more pronounced. Alas, the cross‐sectional design precludes causal inferences. Another key limitation is the reliance on treatment codes as a proxy for the severity of clinical diagnoses. Overall, this study emphasizes that periodontitis escalating severity may serve as a valuable clinical marker for heightened comorbid disease risk.

## AUTHOR CONTRIBUTIONS

*Conceptualization*: Muhammad H. A. Saleh. *Critical review and final approval*: Muhammad H. A. Saleh, Hamoun Sabri. *Data collection*: Muhammad H. A. Saleh. *Funding*: Muhammad H. A. Saleh. *Manuscript writing*: Muhammad H. A. Saleh, Hamoun Sabri. *Statistical analysis*: Hamoun Sabri.

## CONFLICT OF INTEREST STATEMENT

The authors declare no conflicts of interest regarding this research study.

## Supporting information



Supporting Information

Supporting Information

Supporting Information

Supporting Information

Supporting Information

## Data Availability

Data are not available due to legal/commercial restrictions.

## Footnotes

* RStudio software (RStudio, PBC, Boston, MA, USA)

† nnet, car, glmnet and lme4 (glmer function) packages.
