# Supplementary figures and images for: Dose‐dependent association of systemic comorbidities with periodontitis severity: A large population cross‐sectional study
Source: J Periodontol. 2025 Aug 8;97(2):297–312. doi: 10.1002/JPER.25-0055 (PMC13001135; doi:10.1002/JPER.25-0055)

**Figure S1.** Subject eligibility assessment and selection flowchart.

**
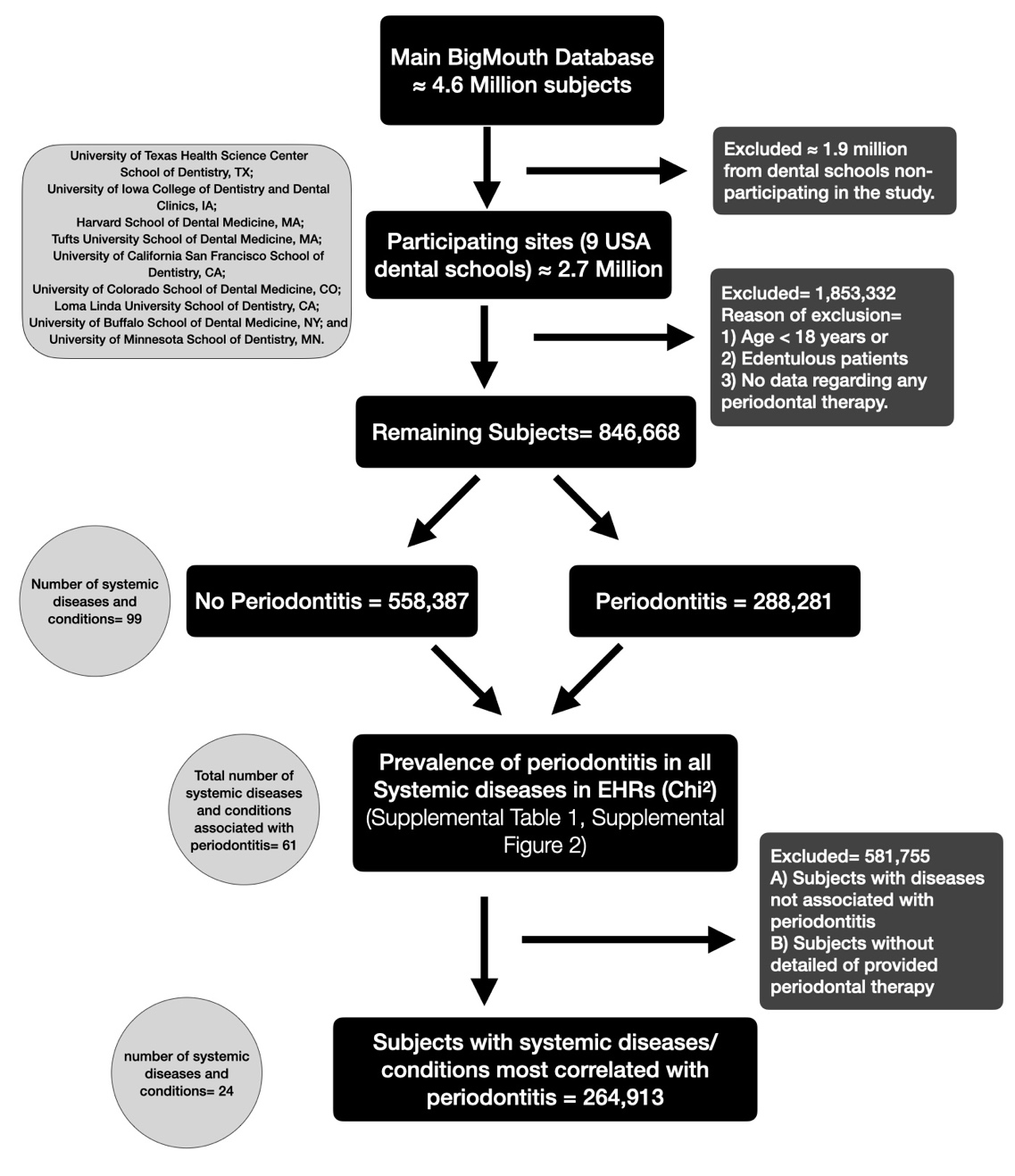
**

Supplement: Supplementary file 1 — Supporting Information [file JPER-97-297-s001.docx]

**Figure S2.** CDT codes used in patient cohort selection.

**
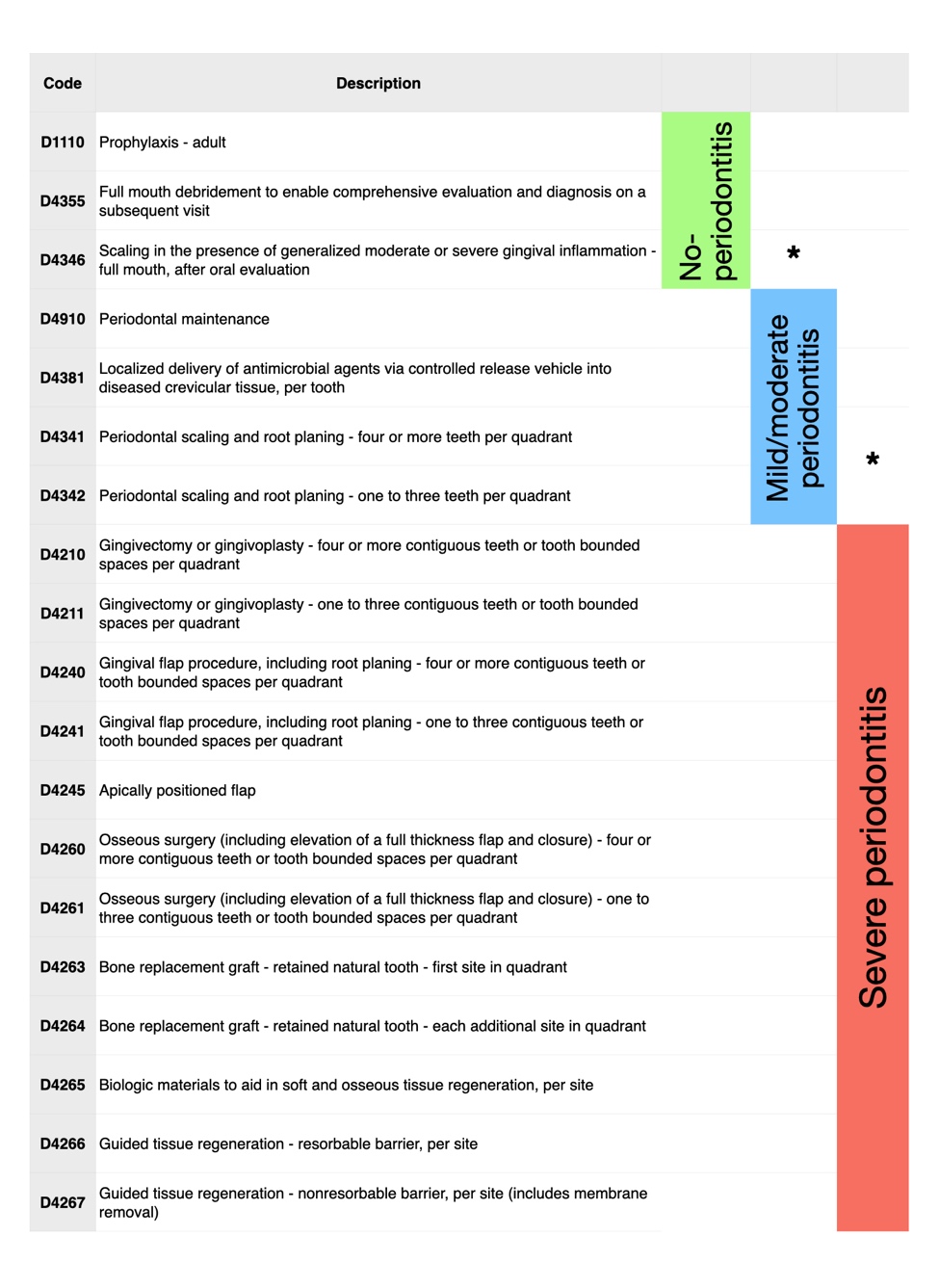
**

Supplement: Supplementary file 2 — Supporting Information [file JPER-97-297-s002.docx]
