# Supplementary material for: Dose‐dependent association of systemic comorbidities with periodontitis severity: A large population cross‐sectional study
Source: J Periodontol. 2025 Aug 8;97(2):297–312. doi: 10.1002/JPER.25-0055 (PMC13001135; doi:10.1002/JPER.25-0055)
Supplement: Supplementary file 3 — Supporting Information [file JPER-97-297-s004.docx]

**Table S1.** Initial chi-squared test results in assessment of eligible systemic conditions to include in final analysis.

|  | **TOTAL**  **(N)** | **PERIODONTAL HEALTH (N)** |  | **MALE**  **(%)** | **FEMALE (%)** | **PERIODONTITIS (N)** |  | **MALE (%)** | **FEMALE (%)** | **P VALUE** | **SIGNIFICANCE** | | |
| --- | --- | --- | --- | --- | --- | --- | --- | --- | --- | --- | --- | --- | --- |
|  | **846,668** | **558,387** | 65.95% | 44.4% | 55.6% | **288,281** | 34.05% | 49.16% | 50.84% |  | **Significant (<0.05)** | **Significant (<0.01)** | **Significant (<0.001)** |
| **Smoking** | 70464 | 41406 | 58.76 | 21882 | 19374 | 29058 | 41.24 | 16808 | 12133 | 0.00000000000000022000 | Y | Y | Y |
| **Breast cancer** | 6873 | 3913 | 56.93 | 87 | 3820 | 2960 | 43.07 | 99 | 2856 | 0.00000000000000022000 | Y | Y | Y |
| **Colon cancer** | 6550 | 3397 | 51.86 | 1473 | 1920 | 3153 | 48.14 | 1695 | 1457 | 0.00000000000000022000 | Y | Y | Y |
| **Family history of cancers** | 28060 | 17799 | 63.43 | 6087 | 11613 | 10261 | 36.57 | 3864 | 6352 | 0.00000000000000022000 | Y | Y | Y |
| **Malignant Cancers** | 12928 | 7877 | 60.93 | 3374 | 4491 | 5051 | 39.07 | 2532 | 2507 | 0.00000000000000022000 | Y | Y | Y |
| **Heart attack** | 5614 | 3369 | 60.01 | 1952 | 1413 | 2245 | 39.99 | 1489 | 750 | 0.00000000000000022000 | Y | Y | Y |
| **High blood pressure** | 59358 | 34047 | 57.36 | 15967 | 18006 | 25311 | 42.64 | 13269 | 11979 | 0.00000000000000022000 | Y | Y | Y |
| **Anorexia** | 249 | 178 | 71.49 | 28 | 150 | 71 | 28.51 |  |  | 0.00000000000000022000 | Y | Y | Y |
| **Diabetes** | 24528 | 13890 | 56.63 | 6385 | 7483 | 10638 | 43.37 | 5719 | 4887 | 0.00000000000000022000 | Y | Y | Y |
| **Hypothyroidism** | 8220 | 4966 | 60.41 | 941 | 4009 | 3254 | 39.59 | 772 | 2472 | 0.00000000000000022000 | Y | Y | Y |
| **Hearing impairment** | 11998 | 7313 | 60.95 | 3627 | 3673 | 4685 | 39.05 | 2664 | 2009 | 0.00000000000000022000 | Y | Y | Y |
| **Vision impairment** | 12756 | 7616 | 59.71 | 2879 | 4695 | 5140 | 40.29 | 2205 | 2910 | 0.00000000000000022000 | Y | Y | Y |
| **HIV** | 667 | 384 | 57.57 | 315 | 67 | 283 | 42.43 | 244 | 39 | 0.00000000000000022000 | Y | Y | Y |
| **Dialysis** | 785 | 390 | 49.68 | 204 | 182 | 395 | 50.32 | 242 | 150 | 0.00000000000000022000 | Y | Y | Y |
| **Kidney stones** | 4669 | 2749 | 58.88 | 1491 | 1242 | 1920 | 41.12 | 1188 | 726 | 0.00000000000000022000 | Y | Y | Y |
| **Renal failure/insufficiency** | 1411 | 750 | 53.15 | 342 | 404 | 661 | 46.85 | 366 | 291 | 0.00000000000000022000 | Y | Y | Y |
| **Arthritis** | 12799 | 7114 | 55.58 | 2158 | 4922 | 5685 | 44.42 |  |  | 0.00000000000000022000 | Y | Y | Y |
| **Gout** | 1389 | 756 | 54.43 | 501 | 252 | 633 | 45.57 | 455 | 172 | 0.00000000000000022000 | Y | Y | Y |
| **Osteoporosis** | 8024 | 4893 | 60.98 | 521 | 4360 | 3131 | 39.02 | 405 | 2719 | 0.00000000000000022000 | Y | Y | Y |
| **Depression** | 12967 | 8010 | 61.77 | 2510 | 5453 | 4957 | 38.23 | 1842 | 3078 | 0.00000000000000022000 | Y | Y | Y |
| **CVA or TIA** | 4522 | 2667 | 58.98 | 1202 | 1463 | 1855 | 41.02 | 969 | 880 | 0.00000000000000022000 | Y | Y | Y |
| **Asthma** | 19770 | 14236 | 72.01 | 4954 | 9256 | 5534 | 27.99 | 2131 | 3393 | 0.00000000000000022000 | Y | Y | Y |
| **Acute Bronchitis** | 16814 | 11902 | 70.79 | 4599 | 7297 | 4912 | 29.21 | 2279 | 2631 | 0.00000000000000022000 | Y | Y | Y |
| **Emphysema/COPD** | 1994 | 1065 | 53.41 | 492 | 572 | 929 | 46.59 | 471 | 456 | 0.00000000000000022000 | Y | Y | Y |
| **GERD** | 24673 | 15606 | 63.25 | 6059 | 9503 | 9067 | 36.75 | 4189 | 4846 | 0.00000000000000022000 | Y | Y | Y |
| **Hepatitis C** | 1535 | 816 | 53.16 | 483 | 327 | 719 | 46.84 | 478 | 237 | 0.00000000000000022000 | Y | Y | Y |
| **Dry Mouth** | 23737 | 14478 | 60.99 | 5027 | 9395 | 9259 | 39.01 | 3870 | 5348 | 0.00000000000000022000 | Y | Y | Y |
| **Long term Steroids (> 1year)** | 8977 | 5554 | 61.87 | 1913 | 3621 | 3423 | 38.13 | 1376 | 2035 | 0.00000000000000025310 | Y | Y | Y |
| **Chronic bronchitis or emphysema** | 3284 | 1944 | 59.20 | 635 | 1302 | 1340 | 40.80 | 529 | 800 | 0.00000000000000031780 | Y | Y | Y |
| **Cataract** | 3841 | 2295 | 59.75 | 937 | 1356 | 1546 | 40.25 | 702 | 842 | 0.00000000000000050010 | Y | Y | Y |
| **Sleep apnea** | 2664 | 1567 | 58.82 | 923 | 643 | 1097 | 41.18 | 705 | 391 | 0.00000000000000866300 | Y | Y | Y |
| **Rheumatoid arthritis** | 13597 | 8543 | 62.83 | 2875 | 5661 | 5054 | 37.17 | 2130 | 2921 | 0.00000000000001048000 | Y | Y | Y |
| **Glaucoma** | 1865 | 1073 | 57.53 | 482 | 591 | 792 | 42.47 | 392 | 399 | 0.00000000000001930000 | Y | Y | Y |
| **Alcohol use** | 121580 | 79053 | 65.02 | 33277 | 45579 | 42527 | 34.98 | 20593 | 21810 | 0.00000000000014780000 | Y | Y | Y |
| **Thyroid problems** | 16424 | 10408 | 63.37 | 1930 | 8448 | 6016 | 36.63 | 1421 | 4580 | 0.00000000000193600000 | Y | Y | Y |
| **Sinusitis** | 14780 | 10148 | 68.66 | 3441 | 6687 | 4632 | 31.34 | 1759 | 2867 | 0.00000000000250000000 | Y | Y | Y |
| **Marijuana use** | 4385 | 2681 | 61.14 | 1758 | 915 | 1704 | 38.86 | 1231 | 465 | 0.00000000001765000000 | Y | Y | Y |
| **Recreational drug use** | 3651 | 2217 | 60.72 | 1301 | 906 | 1434 | 39.28 | 923 | 505 | 0.00000000002680000000 | Y | Y | Y |
| **Skin cancer** | 5028 | 3096 | 61.58 |  |  | 1932 | 38.42 |  |  | 0.00000000005656000000 | Y | Y | Y |
| **AIDS** | 810 | 449 | 55.43 | 352 | 93 | 361 | 44.57 | 293 | 65 | 0.00000000033090000000 | Y | Y | Y |
| **Hepatitis B** | 829 | 462 | 55.73 | 222 | 237 | 367 | 44.27 | 220 | 144 | 0.00000000065410000000 | Y | Y | Y |
| **Congenital heart defect** | 2327 | 1394 | 59.91 | 594 | 796 | 933 | 40.09 | 437 | 496 | 0.00000000082070000000 | Y | Y | Y |
| **Coronary heart disease** | 3014 | 1678 | 55.67 | 1082 | 591 | 1336 | 44.33 | 953 | 377 | 0.00000000082070000000 | Y | Y | Y |
| **Pacemaker/Implanted defibrillator** | 3228 | 1964 | 60.84 | 1214 | 747 | 1264 | 39.16 | 855 | 408 | 0.00000000094770000000 | Y | Y | Y |
| **Anxiety** | 11267 | 7127 | 63.26 | 2123 | 4964 | 4140 | 36.74 | 1437 | 2676 | 0.00000000129000000000 | Y | Y | Y |
| **Bisphosphonates** | 1935 | 1154 | 59.64 | 103 | 1045 | 781 | 40.36 | 79 | 702 | 0.00000000513300000000 | Y | Y | Y |
| **Arteriosclerosis** | 1044 | 600 | 57.47 | 392 | 206 | 444 | 42.53 | 312 | 131 | 0.00000000877400000000 | Y | Y | Y |
| **Rheumatic heart disease** | 1183 | 690 | 58.33 | 252 | 437 | 493 | 41.67 | 213 | 280 | 0.00000003641000000000 | Y | Y | Y |
| **Hepatitis A** | 786 | 447 | 56.87 | 193 | 252 | 339 | 43.13 | 181 | 156 | 0.00000009431000000000 | Y | Y | Y |
| **Pneumonia** | 3054 | 1880 | 61.56 | 647 | 1227 | 1174 | 38.44 | 462 | 702 | 0.00000031760000000000 | Y | Y | Y |
| **Hyperthyroidism** | 1140 | 670 | 58.77 | 123 | 541 | 470 | 41.23 | 113 | 355 | 0.00000036300000000000 | Y | Y | Y |
| **Heart failure** | 815 | 471 | 57.79 | 199 | 272 | 344 | 42.21 | 180 | 164 | 0.00000105500000000000 | Y | Y | Y |
| **Psoriasis** | 5811 | 3678 | 63.29 | 1559 | 2112 | 2133 | 36.71 |  |  | 0.00001906000000000000 | Y | Y | Y |
| **Syphilis** | 188 | 96 | 51.06 | 56 | 40 | 92 | 48.94 | 56 | 35 | 0.00002326000000000000 | Y | Y | Y |
| **Blood/hematologic disorder** | 3398 | 2127 | 62.60 | 622 | 1497 | 1271 | 37.40 | 447 | 823 | 0.00003825000000000000 | Y | Y | Y |
| **Bulimia** | 219 | 173 | 79.00 | 15 | 157 | 46 | 21.00 | 7 | 39 | 0.00006259000000000000 | Y | Y | Y |
| **Ovarian/Uterine cancer** | 552 | 320 | 57.97 | 6 | 313 | 232 | 42.03 | 4 | 228 | 0.00009119000000000000 | Y | Y | Y |
| **ADD/ADHD** | 2216 | 1549 | 69.90 | 715 | 824 | 667 | 30.10 | 322 | 342 | 0.00009375000000000000 | Y | Y | Y |
| **Iron deficiency anemia** | 5840 | 3713 | 63.58 | 422 | 3266 | 2127 | 36.42 | 330 | 1785 | 0.00013070000000000000 | Y | Y | Y |
| **Cirrhosis/Chronic hepatitis** | 1738 | 1073 | 61.74 | 485 | 588 | 665 | 38.26 | 350 | 313 | 0.00022840000000000000 | Y | Y | Y |
| **Tuberculosis** | 1404 | 864 | 61.54 | 338 | 522 | 540 | 38.46 | 240 | 299 | 0.00053250000000000000 | Y | Y | Y |
| **Bladder cancer** | 94 | 48 | 51.06 | 36 | 12 | 46 | 48.94 | 33 | 13 | 0.00331100000000000000 | Y | Y | N |
| **Angina** | 4594 | 2938 | 63.95 | 1216 | 1717 | 1656 | 36.05 | 799 | 853 | 0.00437000000000000000 | Y | Y | N |
| **Lung cancer** | 238 | 138 | 57.98 | 54 | 83 | 100 | 42.02 | 47 | 52 | 0.01154000000000000000 | Y | N | N |
| **Mononucleosis** | 652 | 461 | 70.71 | 182 | 278 | 191 | 29.29 | 100 | 91 | 0.01168000000000000000 | Y | N | N |
| **Parkinson’s disease** | 286 | 168 | 58.74 | 91 | 76 | 118 | 41.26 | 72 | 46 | 0.01204000000000000000 | Y | N | N |
| **Mitral Valve prolapse** | 2044 | 1400 | 68.49 | 344 | 1055 | 644 | 31.51 | 189 | 454 | 0.01618000000000000000 | Y | N | N |
| **Palpitations** | 1962 | 1246 | 63.51 | 252 | 990 | 716 | 36.49 | 186 | 529 | 0.02359000000000000000 | Y | N | N |
| **History of Endocarditis** | 556 | 341 | 61.33 | 152 | 189 | 215 | 38.67 | 106 | 109 | 0.02414000000000000000 | Y | N | N |
| **Organ transplant** | 1112 | 700 | 62.95 | 323 | 375 | 412 | 37.05 | 223 | 188 | 0.03735000000000000000 | Y | N | N |
| **Dementia/Alzheimer’s** | 421 | 257 | 61.05 | 92 | 165 | 164 | 38.95 | 67 | 97 | 0.03814000000000000000 | Y | N | N |
| **Irritable bowel syndrome** | 1453 | 993 | 68.34 | 244 | 743 | 460 | 31.66 | 153 | 305 | 0.05787000000000000000 | N | N | N |
| **Lymphoma** | 238 | 143 | 60.08 | 72 | 71 | 95 | 39.92 | 49 | 46 | 0.06549000000000000000 | N | N | N |
| **Adrenal Gland disorder** | 270 | 165 | 61.11 | 43 | 122 | 105 | 38.89 | 27 | 78 | 0.10650000000000000000 | N | N | N |
| **Epilepsy** | 4538 | 3044 | 67.08 | 1354 | 1683 | 1494 | 32.92 | 750 | 740 | 0.11170000000000000000 | N | N | N |
| **Crohn’s disease** | 528 | 366 | 69.32 | 152 | 212 | 162 | 30.68 | 79 | 83 | 0.11250000000000000000 | N | N | N |
| **Sickle cell trait** | 316 | 195 | 61.71 | 33 | 160 | 121 | 38.29 | 29 | 91 | 0.12540000000000000000 | N | N | N |
| **Thrombocytopenic Purpura (ITP)** | 168 | 102 | 60.71 | 30 | 71 | 66 | 39.29 | 24 | 42 | 0.17670000000000000000 | N | N | N |
| **Leukemia** | 172 | 105 | 61.05 | 54 | 51 | 67 | 38.95 | 43 | 24 | 0.20160000000000000000 | N | N | N |
| **Active Tuberculosis** | 794 | 507 | 63.85 | 190 | 317 | 287 | 36.15 | 119 | 167 | 0.22620000000000000000 | N | N | N |
| **Bleeding disorders** | 541 | 343 | 63.40 | 136 | 206 | 198 | 36.60 | 86 | 110 | 0.22760000000000000000 | N | N | N |
| **Paget’s disease** | 28 | 15 | 53.57 | 4 | 11 | 13 | 46.43 | 4 | 9 | 0.23680000000000000000 | N | N | N |
| **Low blood pressure** | 664 | 423 | 63.70 | 114 | 308 | 241 | 36.30 | 73 | 167 | 0.23760000000000000000 | N | N | N |
| **Artificial heart valves** | 919 | 589 | 64.09 | 361 | 228 | 330 | 35.91 | 217 | 113 | 0.24790000000000000000 | N | N | N |
| **Hemophilia B** | 82 | 59 | 71.95 | 31 | 28 | 23 | 28.05 | 10 | 13 | 0.30300000000000000000 | N | N | N |
| **Penicillin Allergy** | 30702 | 20324 | 66.20 | 7765 | 12486 | 10378 | 33.80 | 4530 | 5815 | 0.35630000000000000000 | N | N | N |
| **Multiple sclerosis** | 436 | 297 | 68.12 | 64 | 233 | 139 | 31.88 | 26 | 113 | 0.36540000000000000000 | N | N | N |
| **Anemia** | 20881 | 13710 | 65.66 | 6064 | 7629 | 7171 | 34.34 | 3938 | 3219 | 0.36900000000000000000 | N | N | N |
| **Oropharyngeal cancer** | 218 | 137 | 62.84 | 85 | 51 | 81 | 37.16 | 58 | 23 | 0.36990000000000000000 | N | N | N |
| **PTSD** | 1112 | 719 | 64.66 | 230 | 480 | 393 | 35.34 | 155 | 228 | 0.37960000000000000000 | N | N | N |
| **Hepatitis** | 17158 | 11369 | 66.26 | 4682 | 6672 | 5789 | 33.74 | 2992 | 2788 | 0.39190000000000000000 | N | N | N |
| **Thalassemia** | 355 | 227 | 63.94 | 68 | 157 | 128 | 36.06 | 48 | 79 | 0.45790000000000000000 | N | N | N |
| **Hemophilia A** | 147 | 93 | 63.27 | 51 | 42 | 54 | 36.73 | 33 | 21 | 0.54840000000000000000 | N | N | N |
| **Sickle cell disease** | 94 | 59 | 62.77 | 19 | 40 | 35 | 37.23 | 11 | 24 | 0.58720000000000000000 | N | N | N |
| **Multiple myeloma** | 45 | 28 | 62.22 | 13 | 15 | 17 | 37.78 | 12 | 5 | 0.71090000000000000000 | N | N | N |
| **Gastrointestinal (GI) disorder** | 4881 | 3228 | 66.13 | 1263 | 1959 | 1653 | 33.87 | 696 | 954 | 0.79850000000000000000 | N | N | N |
| **STDs** | 5477 | 3603 | 65.78 | 1550 | 2040 | 1874 | 34.22 | 955 | 907 | 0.80470000000000000000 | N | N | N |
| **Lupus** | 832 | 546 | 65.63 |  |  | 286 | 34.38 |  |  | 0.87130000000000000000 | N | N | N |
| **Hepatitis D** | 19 | 12 | 63.16 | 6 | 6 | 7 | 36.84 | 4 | 3 | 0.98810000000000000000 | N | N | N |
