# Supplementary material for: Dose‐dependent association of systemic comorbidities with periodontitis severity: A large population cross‐sectional study
Source: J Periodontol. 2025 Aug 8;97(2):297–312. doi: 10.1002/JPER.25-0055 (PMC13001135; doi:10.1002/JPER.25-0055)
Supplement: Supplementary file 4 — Supporting Information [file JPER-97-297-s003.docx]

**Table S2.** Summary of Model Fit Statistics (AIC, BIC, R²). This table presents the mean and range (Min–Max) of Akaike Information Criterion (AIC), Bayesian Information Criterion (BIC), and McFadden’s pseudo-R² values across all regressions within each model type. These measures provide an assessment of model fit and the robustness of the multinomial logistic regression analyses.

| **Model** | **Mean AIC** | **AIC Range (Min-Max)** | **Mean BIC** | **BIC Range (Min-Max)** | **Mean McFadden's R²** | **R² Range (Min-Max)** |
| --- | --- | --- | --- | --- | --- | --- |
| **Model 1 (Unadjusted)** | 259607.5 | 251896.0 - 269883.0 | 260138.1 | 250938.0 - 269650.0 | 0.1272 | 0.101 -0.154 |
| **Model 2 (Adjusted for Age & Sex)** | 250045.65 | 240662.0 - 258248.0 | 252997.9 | 241898.0 - 259870.0 | 0.1731 | 0.142- 0.198 |
| **Model 3 (Fully Adjusted)** | 239509 | 230233.0 - 249268.0 | 238740.2 | 231198.0- 250494.0 | 0.2076 | 0.173-0.235 |
