# Supplementary material for: Dose‐dependent association of systemic comorbidities with periodontitis severity: A large population cross‐sectional study
Source: J Periodontol. 2025 Aug 8;97(2):297–312. doi: 10.1002/JPER.25-0055 (PMC13001135; doi:10.1002/JPER.25-0055)
Supplement: Supplementary file 5 — Supporting Information [file JPER-97-297-s005.docx]

**Supplementary Material (results and discussion)**

**Study title:** Dose-Dependent Association of systemic comorbidities with periodontitis severity: A large population cross‐sectional study.

**Authors:** Muhammad H.A Saleh, Hamoun Sabri

**3. Results**

**3.2.1. Model 1 (unadjusted model)**

The unadjusted regression analysis (Model 1) demonstrated significant associations between various factors and the likelihood of mild/moderate and severe periodontitis. Age was a strong predictor, with each year of increase associated with higher odds of mild/moderate periodontitis (OR: 1.04, 95% CI: 1.04–1.04, p < 0.0001) and severe periodontitis (OR: 1.05, 95% CI: 1.04–1.05, p < 0.0001). Males were more likely to develop mild/moderate (OR: 1.42, 95% CI: 1.40–1.44, p < 0.0001) and severe periodontitis (OR: 1.46, 95% CI: 1.32–1.61, p < 0.0001) compared to females. Smoking was strongly associated with mild/moderate (OR: 1.95, 95% CI: 1.91–1.99, p < 0.0001) and severe periodontitis (OR: 5.69, 95% CI: 5.15–6.29, p < 0.0001), while alcohol consumption showed weaker associations (mild/moderate OR: 1.06, 95% CI: 1.04–1.08, p < 0.0001; severe OR: 1.23, 95% CI: 1.11–1.36, p = 0.00005). Diabetes and cardiovascular disease were highly significant predictors, with diabetes showing ORs of 3.47 (95% CI: 3.35–3.60, p < 0.0001) for mild/moderate and 8.76 (95% CI: 7.72–9.95, p < 0.0001) for severe periodontitis, and cardiovascular disease associated with mild/moderate (OR: 3.12, 95% CI: 3.03–3.20, p < 0.0001) and severe periodontitis (OR: 5.73, 95% CI: 5.11–6.42, p < 0.0001). Other conditions, such as hypothyroidism (mild/moderate OR: 2.10, 95% CI: 1.97–2.25, p < 0.0001; severe OR: 1.98, 95% CI: 1.37–2.85, p = 0.00025), arthritis (mild/moderate OR: 3.13, 95% CI: 2.99–3.29, p < 0.0001; severe OR: 4.50, 95% CI: 3.70–5.48, p < 0.0001), and dementia (mild/moderate OR: 2.63, 95% CI: 2.39–2.88, p < 0.0001; severe OR: 6.33, 95% CI: 4.65–8.62, p < 0.0001), were also significantly associated with periodontitis severity. Skin cancer demonstrated a particularly strong association with severe periodontitis (OR: 74.61, 95% CI: 54.85–101.50, p < 0.0001).

**3.2.2. | Model 2 (adjusted for age and sex) [supplementary material]**

The second regression model, adjusted for age and sex, revealed that several factors remained significantly associated with the likelihood of mild/moderate and severe periodontitis, though some associations were attenuated compared to the unadjusted model. Smoking continued to exhibit a strong association with periodontitis severity, with adjusted ORs of 1.78 (95% CI: 1.74–1.82, p < 0.001) for mild/moderate and 5.21 (95% CI: 4.71–5.76, p < 0.001) for severe periodontitis. Alcohol consumption also remained significantly associated, with mild/moderate and severe ORs of 1.07 (95% CI: 1.05–1.09, p < 0.001) and 1.24 (95% CI: 1.12–1.37, p < 0.01), respectively. Cardiovascular disease retained its strong association with mild/moderate periodontitis (OR: 1.74, 95% CI: 1.69–1.79, p < 0.001) and severe periodontitis (OR: 3.26, 95% CI: 2.88–3.69, p < 0.001). Similarly, diabetes was independently associated with mild/moderate (OR: 2.20, 95% CI: 2.12–2.29, p < 0.001) and severe periodontitis (OR: 5.59, 95% CI: 4.90–6.39, p < 0.001).

Other systemic conditions, including arthritis (mild/moderate OR: 1.75, 95% CI: 1.66–1.84, p < 0.001; severe OR: 2.37, 95% CI: 1.94–2.91, p < 0.001), hypertension (mild/moderate OR: 1.97, 95% CI: 1.91–2.03, p < 0.001; severe OR: 3.41, 95% CI: 3.01–3.87, p < 0.001), renal failure/insufficiency (mild/moderate OR: 1.99, 95% CI: 1.68–2.35, p < 0.01; severe OR: 3.93, 95% CI: 2.32–6.67, p < 0.01), dry mouth (mild/moderate OR: 1.78, 95% CI: 1.71–1.86, p < 0.001; severe OR: 2.32, 95% CI: 1.91–2.82, p < 0.001), and heart attack (mild/moderate OR: 1.59, 95% CI: 1.47–1.72, p < 0.001; severe OR: 4.61, 95% CI: 3.67–5.78, p < 0.001) also remained significant predictors. Additionally, sleep apnea (mild/moderate OR: 2.17, 95% CI: 2.04–2.31, p < 0.001; severe OR: 3.52, 95% CI: 2.80–4.43, p < 0.001), anorexia (mild/moderate OR: 0.49, 95% CI: 0.26–0.91, p = 0.02; severe OR: 0.03, 95% CI: 0.03–0.03, p < 0.001), emphysema/COPD (mild/moderate OR: 1.55, 95% CI: 1.33–1.79, p < 0.001; severe OR: 2.31, 95% CI: 1.34–3.97, p = 0.02), HIV (mild/moderate OR: 2.48, 95% CI: 2.12–2.90, p < 0.001; severe OR: 5.42, 95% CI: 3.30–8.88, p < 0.001), hepatitis C (mild/moderate OR: 2.18, 95% CI: 1.75–2.73, p < 0.001; severe OR: 5.69, 95% CI: 2.97–10.89, p < 0.001), and dementia/Alzheimer’s disease (mild/moderate OR: 2.16, 95% CI: 1.96–2.38, p < 0.001; severe OR: 5.12, 95% CI: 3.75–6.98, p < 0.001) maintained significant associations. Notably, skin cancer showed a persistently high OR for severe periodontitis (OR: 35.46, 95% CI: 25.84–48.66, p < 0.001).

Several variables that were significant in Model 1 lost significance in Model 2. Bronchitis, which was associated with mild/moderate periodontitis in Model 1 became non-significant after adjustment for age and sex (p = 0.99 for severe periodontitis). Similarly, hypothyroidism lost significance for severe periodontitis.

**3.3. Model fit statistics**

**Table S2** summarizes the model fit statistics across all regressions. The AIC and BIC values decreased with additional adjustments, indicating improved model performance. McFadden’s R² values ranged from 0.127 to 0.208, suggesting moderate to strong explanatory power, with the fully adjusted model showing the best fit. These results support the robustness of the multinomial logistic regression models used in the analysis.

**4. Discussion**

**4.2. factors with positive associations with periodontitis**

Firstly, when it comes to patient demographics, older age (OR = 1.04 for mild/moderate and OR = 1.05 for severe periodontitis) and male gender (OR = 1.42 for mild/moderate and OR = 1.46 for severe periodontitis) were significantly associated with an increased likelihood of periodontitis. These two are among the most investigated risk determinants in association with periodontitis. A study by Liu et al.,^1^ analyzing 2009-2014 NHANES cycles indicated an OR of 2.55 [2.2–2.96, p< 0.0001] and 3.36 [2.91-3.88, p<0.001] for having moderate-severe periodontitis in individuals between 45-60 and older than 60 compared to younger individuals. Moreover, they reported an OR of 0.52 [0.46-0.58, p<0.0001] for females compared to males on the same outcome. Other studies have also shown results in line with our study^2-4^. In summary, the current evidence provides strong support on age and gender serving as two main risk determinants of periodontitis.

After adjusting for age and gender (Model 2), smoking (OR = 1.78 for mild-moderate and OR = 5.21 for severe periodontitis) and diabetes (OR = 2.20 for mild-moderate and OR = 5.59 for severe periodontitis) remained significant, reinforcing their status as well-established risk factors for periodontitis, as supported by extensive prior research^5, 6^. Among the other systemic conditions that have been shown to have associations with periodontitis, cardiovascular disease (mild-moderate OR: 1.525 [1.480 - 1.572], severe OR: 2.214 [1.939 - 2.528], P<0.001), arthritis (mild-moderate OR: 1.505 [1.430 - 1.584], severe OR: 1.539 [1.248 - 1.897], P<0.001), hypertension (mild-moderate OR: 1.726 [1.674 - 1.779], severe OR: 2.274 [1.986 - 2.605], P<0.001), history of heart attack (mild-moderate OR: 1.306 [1.203 - 1.417], severe OR: 2.824 [2.238 - 3.564], P<0.001) and renal failure/insufficiency (mild/moderate OR: 1.523 [1.281 - 1.811], severe OR: 2.143 [1.261 - 3.642], P<0.01), all significantly associated with periodontitis in both unadjusted and fully adjusted regression models. While these findings align with the extensive evidence suggesting a strong relationship between periodontitis and cardiovascular health, the majority of the existing literature focuses on periodontal disease as a potential contributor to cardiovascular outcomes^7^. A recent umbrella meta-analysis on 57 meta-analyses^8^ revealed that periodontal disease may increase the risk of cardiovascular disease, including coronary heart disease [relative risk (RR) = 1.20, 95% CI 1.12 − 1.29], myocardial infarction (RR = 1.13, 95% CI 1.04 − 1.21), atrial fibrillation or atrial flutter (RR = 1.33, 95% CI 1.29 − 1.38), carotid atherosclerosis (OR = 1.27, 95% CI 1.14 − 1.41), and hypertension (OR = 1.22, 95% CI 1.10 − 1.35). However, direct evidence assessing cardiovascular conditions as risk factors for periodontitis remains limited. Our findings provide new insights into the potential role of cardiovascular health in periodontitis severity. While these associations suggest a possible systemic influence, the mechanisms underlying this relationship require further exploration. Shared risk factors, such as smoking, systemic inflammation, and lifestyle factors, may partially explain these results.

Moreover, a bi-directional relationship between periodontitis and arthritis has been suggested^9^. While a strong evidence supports negative impact of periodontitis on rheumatoid arthritis severity^10^, the opposite direction lacks sufficient evidence to be concluded. Therefore, further longitudinal and case-control studies are needed to explore this association. Sufficient evidence also confirms renal insufficiency being among the risk indicators of periodontitis^11-13^. A recent meta-analysis on 17 studies discovered a significant association between chronic kidney disease (CKD) and periodontitis (OR: 2.36, [95% C.I. 1.25, 4.44]; p = 0.008)^11^. Similarly, another meta-analysis indicated an OR = 2.26 for severe periodontitis [1.69-3.01] when the model was adjusted for risk factors of CKD^12^. This evidence underscores CKD as a significant risk indicator for periodontitis, highlighting the need for integrated management of these coexisting conditions.

On the other hand, several systemic diseases presented associations only with one of mild-moderate or severe categories of periodontitis. Bronchitis (mild-moderate OR:1.409 [1.216 - 1.634], P<0.001), stroke/TIA (mild-moderate OR: 1.663 [1.454 - 1.901], P<0.001). These findings suggest that these conditions may influence the earlier stages of periodontal disease progression but not necessarily its more severe forms. For example, bronchitis and stroke/TIA could be associated with systemic inflammation or vascular changes that exacerbate mild to moderate periodontal inflammation without being strongly linked to the more destructive processes characteristic of severe periodontitis. Alternatively, these associations might reflect differences in the underlying pathophysiology, patient characteristics, or shared risk factors between the two periodontitis categories. Another potential explanation is related to the differences in sample sizes between the mild-moderate and severe periodontitis groups. Further studies are needed to investigate whether these conditions primarily act as contributors to the initial stages of periodontitis development or whether their associations are driven by shared confounders.

Other positive associations in our analysis included: dry mouth (mild-moderate OR: 1.582 (1.513 - 1.655), severe OR: 1.620 [1.324 - 1.982], P< 0.001), sleep apnea (mild-moderate OR: 1.880 [1.766 - 2.002], severe OR: 2.266 [1.791 - 2.869], P<0.001), dementia/Alzheimer’s disease (mild-moderate OR: 1.840 [1.666 - 2.032], severe OR: 3.200 [2.330 - 4.394], P<0.001) as well as viral infections such as HIV (mild-moderate OR: 2.247 [1.921 - 2.628], severe OR: 4.065 [2.463 - 6.709], P<0.001) and hepatitis C (mild-moderate OR: 1.883 [1.500 - 2.364], severe OR: 3.615 [1.871 - 6.986], P<0.0001). The literature remains inconclusive regarding xerostomia (dry mouth), both in the context of non-syndromic cases^14^ and those associated with Sjögren’s syndrome^15^ . However, it has been suggested that xerostomia may indirectly contribute to periodontal inflammation through mechanisms such as increased dental plaque accumulation and alterations in salivary gland function^14^, warranting further research to clarify these pathways. The relationship between dementia and periodontitis has also been investigated throughout the years^16-18^. In the context of dementia/Alzheimer’s disease, the significant associations observed in this study highlight potential mechanisms through which cognitive decline may exacerbate periodontal disease. Dementia can impair an individual’s ability to maintain adequate oral hygiene due to memory loss, reduced motor coordination, or decreased awareness of oral health needs. Additionally, neurodegenerative conditions like Alzheimer’s are associated with systemic inflammation, which may amplify periodontal tissue destruction. The observed stronger association with severe periodontitis (OR: 3.200) suggests that advanced cognitive impairment may disproportionately impact the progression of periodontal disease. Future longitudinal studies are needed to confirm these associations and explore potential interventions to mitigate the impact of dementia on periodontal health. Lopes Pereira et al., in a recent study demonstrated that HIV positive individuals showed an OR of 4.84 (2.65-8.86; P=0.001) for periodontitis^19^. Similarly, an NHANES analysis by Fang et al., indicated an OR of 1.51 (1.29-1.77) for moderate to severe and OR of 1.46 (1.26-1.7) for all periodontitis categories combined for hepatitis C positive individuals^20^. The same study, however, indicated no significant relationship between HIV+ and periodontitis. The evidence on viral infections and periodontitis, though slightly inconsistent, generally indicates a significant association between viral infections like HIV and HCV and more severe forms of periodontitis^19-22^.

**4.4. Other factors**

Several studies have explored the relationship between periodontitis and cancer, primarily focusing on periodontitis as a potential risk factor for cancer. For instance, Ma et al.,^23^ found periodontitis to increase the risk of melanoma with a hazard ratio (HR) of 1.21 (95% CI: 1.03–1.42). Other studies, such as Banthia et al.,^24^ and Xiong et al.,^25^ have suggested mechanisms like systemic inflammation, oxidative stress, and immune dysregulation to explain this Periodontitis → cancer pathway. In contrast, our study examined cancer → Periodontitis, with the latter as the outcome. This distinction makes direct comparison with these studies infeasible. While factors like immune suppression from cancer treatments, systemic inflammation, and behavioral changes may explain the observed associations, there is no direct evidence assessing this pathway. Further longitudinal studies are needed to explore these mechanisms. Our analysis, supported by a large sample size may have amplified the observed effect (severe OR: 31.644 [22.794 - 43.931], P<0.001) necessitating cautious interpretation. These findings do not imply causality or definitive association but emphasize the need for future research to clarify these relationships.

**4.5. Clinical and future research implications**

1. This study confirms the previously established risk factors of periodontitis, such as smoking and diabetes, as well as the risk determinants of age and gender. Consistent with existing evidence, older age and male gender were strongly associated with periodontitis; while smoking and diabetes were reinforced as significant modifiable risk factors influencing disease severity. Furthermore, conditions such as cardiovascular disease, hypertension, and arthritis were corroborated as risk indicators of periodontitis, reflecting their associations without established causality.
2. In addition to confirming these well-studied factors, our analysis identified novel associations with conditions that have limited or mixed evidence in the literature, including asthma, anorexia, and hypothyroidism. These conditions exhibited negative associations with periodontitis severity, suggesting complex and potentially protective mechanisms that require further investigation. Similarly, systemic conditions like HIV, hepatitis C, and dementia/Alzheimer’s disease were strongly associated with severe periodontitis, underlining the need for deeper exploration into the role of systemic inflammation and immune modulation in periodontal health.
3. Skin cancer presented a particularly striking association with severe periodontitis. While this may reflect systemic effects of cancer progression or its treatments, the lack of existing research on this pathway highlights the need for future studies to assess its validity and underlying mechanisms.
4. Clinically, these findings emphasize the importance of a multidisciplinary approach to periodontal care, particularly for patients with systemic conditions. Integrating periodontal screening into the routine management of high-risk populations could improve overall health outcomes.
5. Future research should prioritize longitudinal studies to explore causality and disentangle the complex interplay between systemic conditions and periodontitis. Additionally, mechanistic studies are needed to validate novel associations and investigate how systemic diseases influence periodontal health through inflammatory or immune-mediated pathways. While the large sample size of this study enhances its generalizability, caution is warranted in interpreting the results, as they do not establish causality but provide a basis for hypothesis generation and further research.

**4.6. Limitations**

The limitations of this study should also be acknowledged, and caution is advised when interpreting its results. Firstly, while the exceptionally large sample size of this study offers a first-of-its-kind analysis on such a substantial dataset, it also allows for increased statistical power, enabling the detection of associations that might otherwise go unnoticed in smaller studies. However, the reliance on secondary data introduces potential limitations related to selection bias, variability in data collection methods across clinical centers, and the use of binary disease outcomes, which oversimplify conditions without accounting for their severity or progression. Additionally, systemic conditions were self-reported, which introduces the possibility of recall bias and misclassification, particularly for asymptomatic or undiagnosed cases. Although all treatment planning in participating institutions is conducted under faculty supervision to ensure a standardized level of oversight, the BigMouth dataset provided to researchers does not include center-specific identifiers, preventing the ability to assess inter-institutional variability in diagnostic criteria or treatment approaches. As a result, potential differences in case classification across institutions cannot be ruled out.

The study's cross-sectional design precludes establishing temporal or causal relationships, limiting the ability to infer whether systemic conditions contribute to periodontitis or vice versa. Furthermore, the absence of detailed information on confounding factors, such as oral hygiene practices, dietary habits, or genetic predispositions, may influence the observed associations and residual confounding cannot be ruled out. Additionally, key variables such as socioeconomic status, race/ethnicity, medication intake and oral hygiene habits were not available in the dataset, which may have influenced the observed associations. Future studies should aim to incorporate these factors to provide a more comprehensive analysis of potential confounders. While the dataset's size enhances generalizability, its clinical origin may result in a population skewed toward individuals actively seeking dental care, which could limit applicability to broader populations. Future studies should consider prospective designs or validation cohorts to further enhance the robustness of these findings.

REFERENCES

1. Liu Y, Yu Y, Nickel JC, Iwasaki LR, Duan P, Simmer-Beck M, Brown L. Gender differences in the association of periodontitis and type 2 diabetes. *Int Dent J* 2018;68:433-440.

2. Ioannidou E. The Sex and Gender Intersection in Chronic Periodontitis. *Front Public Health* 2017;5:189.

3. Eke PI, Thornton-Evans GO, Wei L, Borgnakke WS, Dye BA, Genco RJ. Periodontitis in US Adults: National Health and Nutrition Examination Survey 2009-2014. *J Am Dent Assoc* 2018;149:576-588.e576.

4. Eke PI, Wei L, Borgnakke WS, et al. Periodontitis prevalence in adults ≥ 65 years of age, in the USA. *Periodontol 2000* 2016;72:76-95.

5. Genco RJ, Borgnakke WS. Diabetes as a potential risk for periodontitis: association studies. *Periodontol 2000* 2020;83:40-45.

6. Palmer RM, Wilson RF, Hasan AS, Scott DA. Mechanisms of action of environmental factors--tobacco smoking. *J Clin Periodontol* 2005;32 Suppl 6:180-195.

7. Schulze-Späte U, Wurschi L, van der Vorst EPC, Hölzle F, Craveiro RB, Wolf M, Noels H. Crosstalk between periodontitis and cardiovascular risk. *Front Immunol* 2024;15:1469077.

8. Huang D, Wang YY, Li BH, Wu L, Xie WZ, Zhou X, Ma B. Association between periodontal disease and systemic diseases: a cross-sectional analysis of current evidence. *Mil Med Res* 2024;11:74.

9. Hussain SB, Botelho J, Machado V, et al. Is there a bidirectional association between rheumatoid arthritis and periodontitis? A systematic review and meta-analysis. *Semin Arthritis Rheum* 2020;50:414-422.

10. Qiao Y, Wang Z, Li Y, Han Y, Zhou Y, Cao X. Rheumatoid arthritis risk in periodontitis patients: A systematic review and meta-analysis. *Joint Bone Spine* 2020;87:556-564.

11. Serni L, Caroti L, Barbato L, Nieri M, Serni S, Cirami CL, Cairo F. Association between chronic kidney disease and periodontitis. A systematic review and metanalysis. *Oral Dis* 2023;29:40-50.

12. Deschamps-Lenhardt S, Martin-Cabezas R, Hannedouche T, Huck O. Association between periodontitis and chronic kidney disease: Systematic review and meta-analysis. *Oral Dis* 2019;25:385-402.

13. Wu H, Wang S, Wei Z. Periodontitis and risk of mortality in patients with chronic kidney disease: A systematic review with meta-analysis. *J Periodontal Res* 2024;59:868-876.

14. Mizutani S, Ekuni D, Tomofuji T, et al. Relationship between xerostomia and gingival condition in young adults. *J Periodontal Res* 2015;50:74-79.

15. Gheorghe DN, Popescu DM, Dinescu SC, Silaghi M, Surlin P, Ciurea PL. Association between Sjögren's Syndrome and Periodontitis: Epidemiological, Fundamental and Clinical Data: A Systematic Review. *Diagnostics (Basel)* 2023;13.

16. Syrjälä AM, Ylöstalo P, Ruoppi P, Komulainen K, Hartikainen S, Sulkava R, Knuuttila M. Dementia and oral health among subjects aged 75 years or older. *Gerodontology* 2012;29:36-42.

17. Guo H, Chang S, Pi X, Hua F, Jiang H, Liu C, Du M. The Effect of Periodontitis on Dementia and Cognitive Impairment: A Meta-Analysis. *Int J Environ Res Public Health* 2021;18.

18. Ribeiro GR, Costa JL, Ambrosano GM, Garcia RC. Oral health of the elderly with Alzheimer's disease. *Oral Surg Oral Med Oral Pathol Oral Radiol* 2012;114:338-343.

19. Pereira LL, Veiga Siqueira Amorim D, Brito Sampaio W, et al. Factors Associated with Periodontitis in Patients with and without HIV. *Int J Dent* 2023;2023:9929835.

20. Fang T, Liu L, Mao S, Jiang Z, Cao Y, Pan J. Association between virus infection and periodontitis: Evidence from the National Health and Nutrition Examination Survey 2009-2014. *J Med Virol* 2024;96:e29784.

21. Ryder MI. An update on HIV and periodontal disease. *J Periodontol* 2002;73:1071-1078.

22. Chen X, Zeng Z, Xiao L. The association between periodontitis and hepatitis virus infection: a cross-sectional study utilizing data from the NHANES database (2003–2018). *Public Health* 2024;226:114-121.

23. Ma H, Zheng J, Li X. Potential risk of certain cancers among patients with Periodontitis: a supplementary meta-analysis of a large-scale population. *Int J Med Sci* 2020;17:2531-2543.

24. Banthia R, Jain P, Jain AK, Belludi SA, Agarwal N, Patidar M. Evaluation of the association between periodontal disease and total cancer risk: A cross-sectional study. *Dent Med Probl* 2024;61:843-850.

25. Xiong J, Liu H, Li C, Li Y, Feng J. Linking periodontitis with 20 cancers, emphasis on oropharyngeal cancer: a Mendelian randomization analysis. *Sci Rep* 2024;14:12511.
